# Unveiling Host Interactions and Evolutionary Constraints of a Novel Bacteriophage Infecting *Xanthomonas hortorum* pv. *vitians*

**DOI:** 10.1111/1758-2229.70171

**Published:** 2025-10-30

**Authors:** Anaelle Baud, Lucas Morinière, Imane El Idrissi, Fernando Clavijo‐Coppens, Elise Lacroix, Nicolas Taveau, Denis Costechareyre, Franck Bertolla

**Affiliations:** ^1^ Universite Claude Bernard Lyon 1, Laboratoire d'Ecologie Microbienne, UMR CNRS 5557, UMR INRAE 1418, VetAgro Sup Villeurbanne France; ^2^ Greenphage Clapiers France

**Keywords:** bacteriophage, evolutionary trade‐off, LPS receptor, phage–host interactions, Tn‐seq, *Xanthomonas hortorum*

## Abstract

Due to limitations in disease management strategies and the impact of climate change, phytopathogenic bacteria are threatening global crop production. *Xanthomonas hortorum* pv. *vitians,* the causal agent of bacterial leaf spot of lettuce, remains difficult to manage due to the lack of efficient treatment options. As an alternative, phage‐based biocontrol offers a promising solution, but its long‐term efficacy depends on a thorough understanding of phage–host interactions and the potential development of bacterial resistance. In this study, we isolated and characterised the lytic bacteriophage Φ*Xhv*‐1, which defines a novel genus within the class *Caudoviricetes*. Using transposon insertion sequencing, we identified 36 bacterial genes essential for phage susceptibility, primarily involved in lipopolysaccharide biosynthesis and surface polysaccharide modifications. Targeted mutagenesis and fluorescence microscopy confirmed that Φ*Xhv*‐1 adsorbs onto specific residues of the LPS O‐antigen side chains. Phage‐resistant mutants exhibited a decreased motility in vitro and a significant reduction in virulence in planta. These findings reveal a strong evolutionary trade‐off between phage resistance and bacterial fitness, suggesting that resistance emergence in the field may be naturally constrained. This study provides new insights into *X. hortorum* pv. *vitians*–phage interactions and supports the development of sustainable phage‐based biocontrol strategies against bacterial leaf spot of lettuce.

## Graphical abstract

A transposon insertion sequencing approach revealed that the novel phage Φ*Xhv*‐1 targets *Xanthomonas hortorum* pv. *vitians* through LPS O‐antigen recognition. Mutations in LPS conferring phage resistance reduce bacterial motility and virulence, highlighting a trade‐off that may naturally constrain resistance development and support the sustainable phage‐based control strategies.

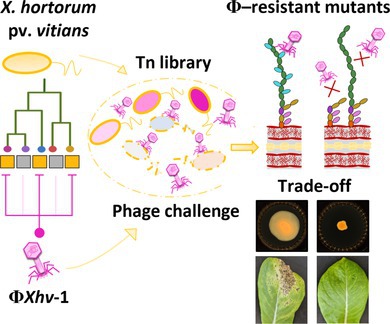

## 1 Introduction

Phytopathogenic bacteria are among the main causes of major damage to crops and yield losses (Savary et al. 2019). In the coming decades, with the predicted aggravation of plant diseases and pests' damages caused by climate changes, and escalating concerns regarding food safety, there is an urgent need to develop new biocontrol‐based integrated plant protection strategies (Singh et al. 2023; Holtappels et al. 2021; Velásquez et al. 2018). Among the 10 main plant‐pathogenic bacterial genera, *Xanthomonas* species cause diseases on almost 400 plant species, some of which are major crops worldwide with critical economic importance (e.g., rice, tomato, cabbage) (Mansfield et al. 2012; Dia et al. 2022). Among these pathogens, *Xanthomonas hortorum* pv. *vitians* is a significant foliar pathogen of cultivated lettuce ( *Lactuca sativa* ) causing a disease known as bacterial leaf spot of lettuce. This disease is present throughout the world and occurs on all types of cultivated lettuces (Koike and Gilbertson 2017). This pathogen has become a concerning threat for lettuce production due to the lack of efficient and practical means of disease control or prevention (Bull and Koike 2005). To address this issue, several studies have investigated the biocontrol potential of *Pseudomonas* spp. Strains (Zboralski et al. 2022) and essential oils from *Origanum* species (Dadasoglu et al. 2011) to inhibit this pathogen. These approaches have shown significant antibacterial activity in vitro and support the development of sustainable and environmentally friendly methods to protect lettuce crops. Further advances in these control strategies are supported by studies on the genetic diversity of *X. hortorum* pv. *vitians*, the molecular mechanisms underlying its adaptation to host plants and key factors driving its pathogenesis (Fayette et al. 2016; Rosenthal et al. 2022; Morinière et al. 2020). Understanding these aspects is crucial for designing targeted biocontrol methods that can effectively limit bacterial infections in the field. In this context, bacteriophage‐based biocontrol is gaining renewed interest (Holtappels et al. 2021; Clavijo‐Coppens et al. 2022).

Lytic bacteriophages (or phages) are considered to be promising alternative bactericides due to their extreme host specificity, self‐replicating nature, biodegradability and non‐toxicity to non‐target organisms (Vila et al. 2024). Furthermore, the use of phages as biocontrol agents against *Xanthomonas* spp. has shown promising results under both greenhouse and field conditions (Nakayinga et al. 2021; Holtappels et al. 2022). The isolation and characterisation of novel phage candidates targeting *X. hortorum* pv. *vitians* strains could open up interesting prospects for the development of an effective biocontrol solution (Boyer et al. 2022). Nevertheless, evaluating the long‐term effectiveness of such a phage therapy solution requires addressing the issue of potential emergence of bacterial resistance, a natural phenomenon occurring during the co‐evolutionary interactions between phages and bacteria. One of the crucial steps in phage infection is the initial attachment of phage particles to specific receptors on the bacterial cell surface, followed by irreversible adsorption, which enables the injection of phage genomic material into the host cell. The bacterial molecular targets required for phage adsorption are diverse and include, in particular, outer membrane proteins, capsular polysaccharide, lipopolysaccharide (LPS) and other bacterial cell appendages, such as flagella and pili (Bertozzi Silva et al. 2016). Alterations of these surface receptors though mutation result in adsorption failure and hence bacterial resistance. To circumvent this, understanding the bacterial molecular targets involved in phage infection is essential for developing effective and sustainable biocontrol strategies. In this context, transposon‐insertion sequencing (Tn‐seq) has emerged as a powerful genomic approach to identify phage susceptibility determinants (Mutalik et al. 2020). Although phage receptors have not been investigated in any known *X. hortorum* pathovar, a recent study in *Xanthomonas campestris* pv. *campestris* identified the involvement of the LPS biosynthetic *wxc* gene cluster as receptors for two newly characterised lytic phages using a transposon insertion (Tn5) library (Holtappels et al. 2022). Since this tripartite molecule, composed of lipid A, a core oligosaccharide and an O‐antigen, also plays critical roles in virulence, resistance mutations often involve trade‐offs. Mutations in the *wxc* cluster confer resistance to the phages FoX2 and FoX6 and also significantly reduce virulence in planta. In *Dickeya solani*, spontaneous phage‐resistant mutants exhibited reduced fitness and virulence on potatoes (Warring et al. 2022). These examples highlight the importance of characterising the bacterial surface structures that serve as phage receptors and understanding how resistance mechanisms affect ecological performance and pathogenic potential.

In this study, we isolated and characterised the lytic phage Φ*Xhv*‐1, which defines a novel genus within the class *Caudoviricetes*. To investigate the host molecular determinants involved in phage susceptibility, a competitive fitness experiment was conducted by infecting a transposon insertion sequencing library previously generated in strain LM16734 (Morinière et al. 2019). Genome‐wide identification of resistance loci was further validated through selective isolation of high‐fitness knock‐out mutants and targeted deletion mutagenesis of functional regions. Insights from these mutants, combined with epifluorescence microscopy of phage adsorption, allowed precise identification of bacterial molecular targets and led us to propose a theoretical model of Φ*Xhv*‐1 adsorption. Finally, we assessed the evolutionary trade‐off associated with phage resistance by evaluating the impact of resistance mutations on bacterial fitness in planta, focusing on symptom development, tissue colonisation and motility. Together, these findings provide new insights into phage–host interactions and the ecological consequences of phage resistance in plant‐associated bacteria.

## 2 Materials and Methods

### 2.1 Bacterial Strains, Plasmids and Growth Conditions

The bacterial strains and plasmids used in this study are listed in Table S1. *X. hortorum* pv. *vitians* strains were routinely cultured on tryptic soy agar (TSA) or broth (TSB) at 28°C. For phage experiments, media were supplemented with CaCl_2_ (1 or 10 mM) to promote adsorption. *Escherichia coli* strains were grown on Lysogeny Broth (LB) medium at 37°C. When required, the media were supplemented with 100 μg mL^−1^ ampicillin, 25 μg mL^−1^ kanamycin, 30 μg mL^−1^ chloramphenicol and 50 μg mL^−1^ for cycloheximide. Sucrose was added at 5% (w/v) final concentration.

### 2.2 Isolation and Amplification of the Phage

Φ*Xhv*‐1 was isolated from wastewater samples collected in Montpellier (France), using an enrichment‐based approach and screened for lytic phages by the double‐layer agar technique, as adapted from Plumet et al. (2023). Filtered effluents were mixed with *X. hortorum* pv. *vitians* culture in middle logarithmic phase. Single clear plaques were purified through two successive re‐isolation steps. Concentrated phage stocks were kept at 4°C in SM buffer (50 mM Tris–HCl [pH 7.5], 8 mM MgSO_4_⸱7H_2_O, 100 mM NaCl). Amplification was performed in liquid cultures with its isolation host *X. hortorum* pv. *vitians* LM16734 at a multiplicity of infection (MOI) of 1. After overnight incubation, cultures were centrifuged and filtered. Phage titres were quantified using standard plaque assays.

### 2.3 Phage Morphological Characterisation by Transmission Electronic Microscopy (TEM)

The preparation of phage samples for TEM observation was performed following the protocol previously described by Plumet et al. (2023), with minor modifications. Briefly, phage particles were concentrated by centrifugation and washed in ammonium acetate buffer. Grids were negatively stained using 2% uranyl acetate and visualised with a JEOL 1400 Flash transmission electron microscope operated at 120 kV.

### 2.4 Whole Genome Sequencing of Bacterial and Phage Genomes, Comparative Genomics

Bacterial DNA was extracted with the Microbial DNA kit (Macherey‐Nagel) and sequenced in Illumina HiSeq 2 × 150 bp (Novogene, UK). Paired‐end reads were assembled in contigs using UNICYCLER v.0.5.0 with a minimum contig size of 200 bp and then annotated with the NCBI RefSeq pipeline. For phylogenomic analysis, a tree was inferred using the Genome BLAST Distance Phylogeny (GBDP) method via the online Type Strain Genome Server (TYGS) (Meier‐Kolthoff and Göker 2019). FastME 2.1.4 was used for tree reconstruction, with a BioNJ starting tree and subtree pruning and regrafting (SPR) post‐processing. Branch support values were calculated from 100 bootstrap replicates. Antiviral defence systems encoded by bacteria were detected with the online tool DefenseFinder (Tesson et al. 2022).

Phage DNA was purified according to Gendre et al. (2022) without the addition of SDS and proteinase K and submitted to MiSeq Illumina sequencing 2 × 150 bp. Reads quality was evaluated with FastQC 0.11.90 (Wingett and Andrews 2018) followed by a quality filtering using PRINSEQ 0.20.4 (Schmieder and Edwards 2011). Reads were assembled using SPAdes 3.15.2 (Nurk et al. 2013). Genome circularisation was performed using UGENE 38.0 (Okonechnikov et al. 2012). The purity of contig sequences was validated using the BLAST software suite (Camacho et al. 2009) against the non‐redundant nucleotide (nt) database. Assembly quality was evaluated with Bowtie2 2.4.4 (Langmead and Salzberg 2012) and Samtools 2.12 (Li et al. 2009). Circular permutations were searched using the MAUVE software 2015‐02‐25 (Darling et al. 2004). BACPHLIP was used to predict the lifestyle of the phage Φ*Xhv*‐1 (Hockenberry and Wilke 2021).

### 2.5 Adsorption Assay

Early‐log‐phase culture of the production strain (OD_600_ ≈ 0.2, 10^8^ CFU mL^−1^) was mixed with Φ*Xhv*‐1 at a MOI of 10^−2^ and incubated at 28°C. Aliquots were collected every 10 min for 1 h, filtered to collect non‐adsorbed (free) phages and quantified by spot assay.

### 2.6 One‐Step Growth Curves

Phage replication dynamics were assessed by infecting early‐exponential phase bacterial cultures with Φ*Xhv*‐1 at a MOI of 0.1. After the adsorption period, unadsorbed phages were removed by centrifugation and washing. Infected cells were then resuspended in fresh TSB and incubated at 28°C under agitation. Aliquots were collected at regular intervals over a 2‐h period for phage titration. Burst size was calculated as the number of phage particles released per infected cell.

### 2.7 Bacterial Growth Inhibition Assay

Early exponential phase cultures of *X. hortorum* pv. *vitians* strains were cocultured with Φ*Xhv*‐1 at a MOI of 1. Growth kinetics were monitored by OD_600_ measurements every 20 min over a 25‐h period using a Bioscreen C MBR BACTERIO (Thermo Fisher Scientific). Control wells without phage were included.

### 2.8 Phage Host Range

The susceptibility of 13 *X. hortorum* pv. *vitians* strains to Φ*Xhv*‐1 was evaluated by growth monitoring (as described above) and spot assays on double‐layer agar plates. The strain panel represented four different geographical origins and reflected the pathovar's genetic diversity, covering all three MLSA phylogenetic groups and including isolates collected between 1949 and 2017 (Table S1). Briefly, each strain was mixed with soft TSB agar (0.6%) supplemented with 1 mM CaCl_2_ and overlaid onto TSA plates. Ten‐microlitre drops of serial dilutions of Φ*Xhv*‐1 (10^10^ PFU mL^−1^) were spotted onto the bacterial lawns and incubated overnight at 28°C. Strains were classified as resistant when the efficiency of plating (EOP) was ≤ 10^−4^. To assess the correlation between bacterial genetic relatedness and Φ*Xhv*‐1 susceptibility, the phylogenetic signal of the sensitive/resistance phenotype (based on EOP values) was evaluated using the delta statistic (*δ*) with a thousand bootstrap replicates as described by Borges et al. (Borges et al. 2019).

### 2.9 Genome‐Wide Phage‐Resistance Genes Screen Using Transposon Insertion Sequencing

A previously generated Tn‐seq mutant library of *X. hortorum* pv. *vitians* LM16734 (Morinière et al. 2019) was co‐cultured with Φ*Xhv*‐1 at a MOI of 1 in TSB supplemented with 10 mM CaCl_2_. After 21 h of co‐culture under agitation, surviving bacterial populations were harvested, and genomic DNA was extracted for transposon insertion sequencing. Control libraries were grown without phage. Tn‐seq library preparation and Illumina sequencing were conducted as previously described (Morinière et al. 2019). In parallel, individual phage‐resistant mutants were isolated from the infected culture and conserved in 30% (w/v) glycerol for downstream analysis. A subset of these was identified by AP‐PCR (Saavedra et al. 2017) and used for further analyses.

### 2.10 Fitness Assessment of Genes Associated With Phage Infection

Sequencing reads pre‐processing for further TnSeq analysis was performed as described previously (Morinière et al. 2019). Read counts were normalised using the “Totreads” method available in TRANSIT v.3.2.1 (DeJesus et al. 2015) to obtain the same total number of reads for each sample. The contribution of each genetic feature to bacterial fitness was determined by performing pairwise comparisons between the phage condition and the control condition with the « Resampling » method available in TRANSIT v.3.2.1. Reads in the 5% N‐terminal and 10% C‐terminal portions of the genetic feature were discarded, and a LOESS (locally estimated scatterplot smoothing) correction for genome positional bias was applied. Genetic features with a log_2_ Fold Change (log_2_FC) > 2 and a *q*‐value ≤ 0.05 were considered critical for successful phage infection.

### 2.11 Construction of *X. hortorum* pv. *vitians* Deletion Mutants

All the primer pairs used in this study are listed in Table S2. Knockout mutants of Regions 2 and 3 of the LPS gene cluster were constructed using the *sacB* counter‐selection system as described previously (Boulanger et al. 2014). Upstream and downstream 650‐bp regions were amplified and cloned into the multiple cloning site (MCS) of the pK18*mobsacB* plasmid using the T5‐Exonuclease‐Dependent‐Assembly (TEDA) method (Xia et al. 2019). All constructions were verified by Sanger sequencing. Plasmids were introduced into *X. hortorum* pv. *vitians* LM16734 by triparental mating with the helper strain *E. coli* RK600 (Ditta et al. 1980). In addition, genomes of the deletion mutants were sequenced.

### 2.12 Phage Adsorption Assay by Fluorescence Microscopy

Phage adsorption on phage‐resistant mutants and the wild type strain LM16734 was assessed by SYBR Gold fluorescence microscopy, as described previously with some modifications (Holtappels et al. 2022). Briefly, phages were fluorescently labelled using SYBR Gold 1X, and excess dye was removed by filtration and concentration. Labelled phages were then incubated with bacterial cultures at a MOI of 400. After 1 h of incubation followed by washing steps, samples were observed under an Axioskop HBO 50 W epifluorescence microscope connected to an Axiocam 503 colour camera (Zeiss). Images were acquired using Zen software with an exposure time of 0.03 s for natural light images and at 0.67 s for fluorescent images (*λ*Ex = 495 nm, *λ*Em = 537 nm).

### 2.13 Pathogenicity Assays of Deletion and Transposon Knock‐Out Mutants

Six Φ*Xhv*‐1‐resistant mutants, including deletion and transposon insertion mutants in the *cps*, LPS1, LPS2 and LPS3 regions, were tested for virulence using both spray inoculation and infiltration assays on lettuce cv. Météore as described previously (Morinière et al. 2020). Disease severity was measured every 2 days on each plant during 3 weeks using our scale disease index. In addition, infiltration assays were conducted on lettuce leaves with bacterial suspensions adjusted to 10^5^ CFU mL^−1^. The bacterial population was enumerated at 0, 2, 7 and 10 days post‐inoculation (DPI). Leaf sampling, sterilisation, homogenisation and CFU quantification were conducted as described previously (Morinière et al. 2022). Bacterial populations were expressed in CFU cm^−2^ of lettuce leaf.

### 2.14 Motility Assay

The same six phage‐resistant mutants tested in virulence assays were assessed for twitching and swarming motility on TSB plates containing 0.6% or 1% agar, respectively. To promote the expression of motility‐associated genes, sucrose was added at a final concentration of 1% (Tian et al. 2021). Bacterial cultures (OD_600_ = 0.8) were centrifuged, and pellets were point‐inoculated onto plates using sterile toothpicks. After 6 days of incubation at 28°C, motility zone diameters were measured using ImageJ software (Schneider et al. 2012).

### 2.15 Data Analysis and Visualisation

All figures were generated using R (version 4.3.3; R Core Team) within RStudio (version 2023.12.1.402 ‘Ocean Storm’ Release; RStudio Team), unless otherwise specified. Visualisations were primarily created using the ggplot2 package (version 3.5.1), with additional packages including readxl (version 1.4.3) for data import, dplyr (version 1.1.4) for data manipulation, ComplexHeatmap (version 2.18.0) and circlize (version 0.4.16) for specific heatmaps, and ggsignif (version 0.6.4) and gridExtra (version 2.3) for statistical annotations and figure arrangement, depending on the figure type.

Additional methodological details are provided in the Supplementary Methods in the Supporting Information.

## 3 Results

### 3.1 Characterisation of a Novel Lytic *Myovirus*‐Like Phage Infecting *X. hortorum* pv. *vitians*

**FIGURE 1:**
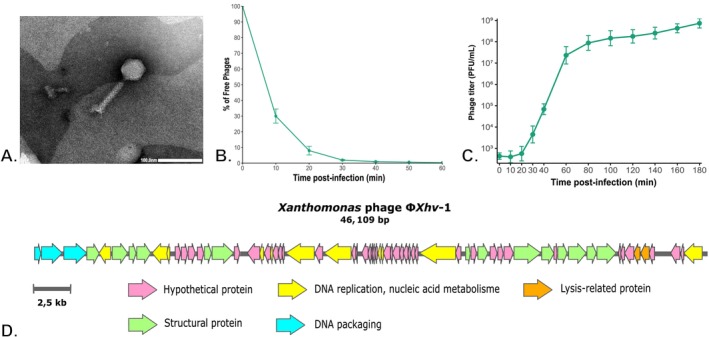
Morphological, functional and genomic characterisation of phage Φ*Xhv*‐1. (A) Transmission electron microscopy (TEM) of Φ*Xhv*‐1 displaying a *myovirus* morphotype with a non‐contracted tail and an icosahedral capsid. Virions were negatively stained with uranyl acetate. Scale bar = 100 nm. (B) Adsorption kinetics of Φ*Xhv*‐1 to *X. hortorum* pv. *vitians* LM16734 at 28°C. The percentage of free (unbound) phages was measured at indicated time points post‐infection. Data points represent the mean ± standard error of the mean (SEM) from three independent experiments. (C) One‐step growth curve (OSGC) of Φ*Xhv*‐1 at 28°C, showing phage titres (PFU mL^−1^) over time. Data represent means ± standard deviation from two independent experiments, each with three technical replicates per time point. (D) Linear genomic map of phage Φ*Xhv*‐1 (46,109 bp), generated with Snapgene (v7.2.1) and edited in Inkscape (v1.4). Arrows indicate the category and direction of coding domain sequences (CDS): (pink) hypothetical protein, (yellow) DNA replication and nucleic acid metabolism, (green) structural protein, (blue) DNA packaging, and (orange) lysis‐related proteins.

Phage Φ*Xhv*‐1 was isolated from a wastewater treatment plant (Montpellier, France) using *X. hortorum* pv. *vitians* LM16734 as the host. Following amplification, the phage reached a titre of 6 × 10^10^ PFU mL^−1^. The phages produced small clear plaques, with diameters of approximately 1.5 ± 0.26 mm on bacterial lawn (Figure S1). TEM micrographs revealed a typical icosahedral capsid with an average diameter of 54.45 ± 0.65 nm, and a medium‐length long contractile tail of approximately 86.35 ± 1.26 nm in length and 14.89 ± 0.37 nm in width (Figure 1A), suggesting a morphology typical of *myoviruses*. Whole‐genome sequencing reported a 46,109‐bp double‐stranded DNA genome with a GC content of 62% (GenBank accession: PV408261). Gene annotation identified 68 coding domain sequences (CDSs) with 33 functionally annotated and 35 remaining hypothetical or unknown (Table S4). The CDSs were categorised into four functional groups: (i) lysis, (ii) structural proteins, (iii) DNA packaging, (iv) DNA replication and nucleic acid metabolism (Figure 1D). No known virulence/toxin, antibiotic resistance or genes associated with a temperate lifestyle were detected. Consistent with this, a lytic lifestyle was predicted with 77.5% probability. The best hits from BLAST analysis correspond to phages infecting *Xanthomonas* spp. The closest phage to Φ*Xhv*‐1 was BsXeu269p/3 (ON996340.1), with 11% query coverage and 78.98% identity. This corresponds to an estimated intergenomic similarity of approximately 50%, which is below the 70% threshold generally used to define genera among tailed phages, according to current ICTV guidelines (Turner et al. 2023). Given this genetic distance and according to ICTV classification criteria, we propose that Φ*Xhv*‐1 defines a new genus within the class *Caudoviricetes*, tentatively named *Xanthorvitianvirus xhv1*.

### 3.2 Φ*Xhv* ‐1 Displays a Narrow Host Spectrum but Shows Highly Lytic Activity on the Host

**FIGURE 2:**
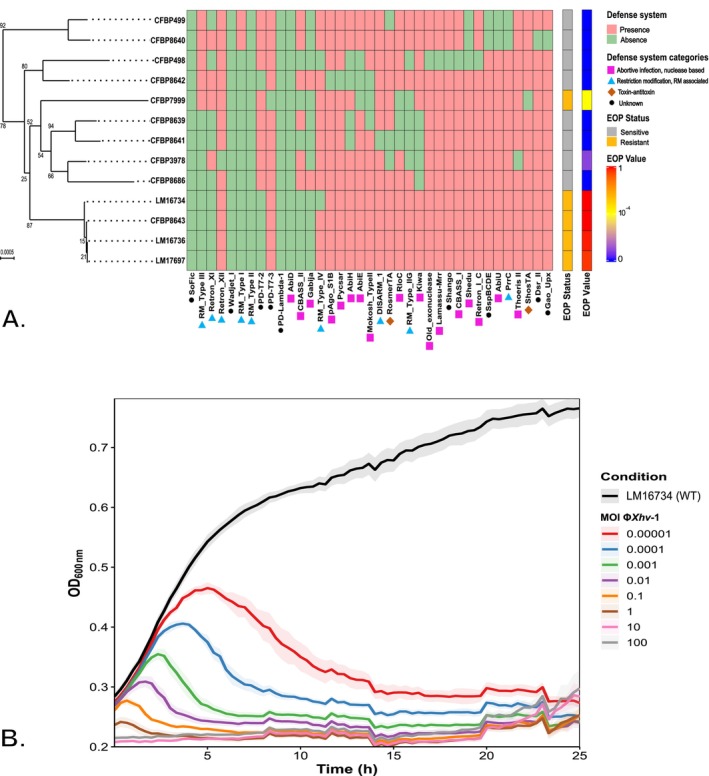
Distribution of bacterial defence systems and phage susceptibility among *X. hortorum* pv. strains. (A) Phylogenetic relationships and defence systems gene repertoire of *X. hortorum* pv. *vitians* strains. The phylogenetic tree on the left was inferred from whole‐genome sequences using TYGS (Meier‐Kolthoff and Göker 2019). The heatmap represents the presence (green) or absence (red) of bacterial defence systems identified with DefenseFinder (Tesson et al. 2022). Symbols below each system name indicate its functional category, as defined by DefenseFinder annotations. Phage Φ*Xhv*‐1 susceptibility is indicated by the efficiency of plating (EOP) status: resistant (grey) or sensitive (yellow). EOP values are represented on a colour scale from blue (low EOP) to red (high EOP), where higher values indicate increased phage infectivity. (B) Growth kinetics of *X. hortorum* pv. *vitians* LM16734 after infection with phage Φ*Xhv*‐1 at various multiplicities of infection (MOI). Optical density at 600 nm was recorded every 20 min for 25 h. The black and coloured curves correspond to the uninfected growth and cultures under MOIs ranging from 0.00001 to 100, respectively. Data represent means ± standard deviation (SD) from three independent replicates.

Among the 13 representative *X. hortorum* pv. *vitians* strains tested (Table S1), Φ*Xhv*‐1 exhibited a narrow host range, infecting only five strains (38%). Four strains (i.e., LM16734, CFBP8643, LM17697 and LM16736) were highly susceptible (EOP ≥ 0.9), and one strain (CFBP7999) displayed intermediate susceptibility (EOP = 3.3 × 10^−4^). The remaining strains were resistant (EOP < 10^−4^) (Figure 2A). These results were confirmed in liquid assays, where no bacterial growth was observed for susceptible strains. To investigate whether Φ*Xhv*‐1 susceptibility correlates with bacterial phylogeny, a phylogenetic signal analysis was performed. This analysis revealed no significant phylogenetic signal (*δ* = 0.46, *p*‐value = 0.27), suggesting that the phage's host range is not correlated with the overall genomic relatedness of the tested strains. A total of 37 distinct anti‐phage defence systems were identified across the 13 genomes of *X. hortorum* pv. *vitians*, with an average of 13 systems per strain. The strain CFBP498 harboured the highest number (*n* = 19), while CFBP8686 carried the fewest (*n* = 9). These systems were classified into functional categories: 17 were identified as abortive infection or nuclease‐based, 9 as restriction‐modification or RM‐associated, 2 as toxin‐antitoxin systems and 9 remained of unknown functional category. To statistically assess the potential correlation between the presence of these defence systems and Φ*Xhv*‐1 susceptibility, a logistic regression analysis was performed. This analysis revealed no significant correlation between the presence of individual anti‐phage defence systems and resistance to Φ*Xhv*‐1 (Figure 2A). In vitro lytic assays on the production strain LM16734 showed that lysis kinetics were MOI dependent. At an MOI of 1, complete lysis of the bacterial population occurred in less than 2 h, while spontaneous resistant mutants appeared after 21 h (Figure 2B). To better characterise Φ*Xhv*‐1 lytic activity on its production strain, key parameters of its infection cycle were determined. The adsorption rate constant was estimated at 1.2 × 10^−7^ mL cell min^−1^, and over 90% of phage particles were adsorbed within the first 20 min (Figure 1B). The latent period was approximately 20 min, followed by a 60 min rise period, leading to a total lytic cycle of 115 min. Each infected cell released an average of 23 newly virions (Figure 1C).

### 3.3 The Tn‐Seq Analysis Unveiled a Set of 36 Genes That Are Essential for the Successful Infection by Φ*Xhv* ‐1

**FIGURE 3:**
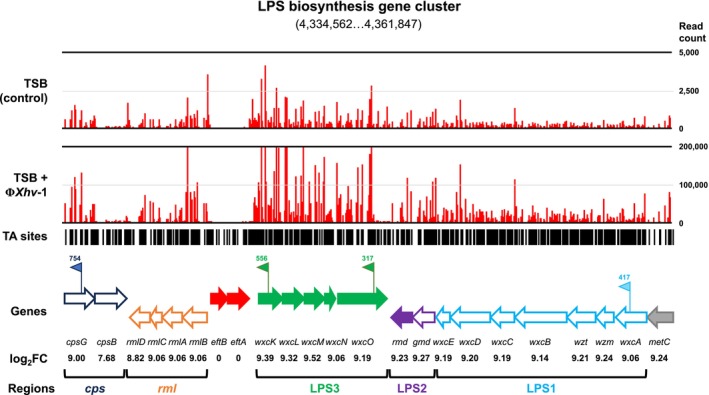
Schematic overview of essential genes involved in phage susceptibility within the LPS biosynthesis gene cluster of *X. hortorum* pv. *vitians* LM16734. Numbers in brackets indicate the width of the viewing window on the LM16734 genome. Black bars represent transposon insertion sites (TA sites) along the locus. Red bar plots (read counts) show the number of sequencing reads mapping to each TA site under control (TSB) and experimental conditions (TSB + Φ*Xhv*‐1). Flag symbols mark the insertion mutants analysed in this study. The number refers to the position of the TA site relative to the gene's start codon, and the flag's orientation indicates the strand of insertion (sense or antisense). The outline colour of each arrow indicates the functional grouping of genes within the LPS biosynthesis cluster, as described by Vorhölter et al. (2001). Red arrows represent essential genes in the in vitro library (Morinière et al. 2019), and open arrows denote genes critical for in planta fitness (Morinière et al. 2022). Gene names and corresponding log_2_FC values are displayed below each gene.

To identify genes involved in the susceptibility of *X. hortorum* pv. *vitians* LM16734 to phage Φ*Xhv*‐1, we used a previously developed Mariner‐based Tn‐seq mutant library (Morinière et al. 2019). After 21 h of coculture with the phage, mutants with Himar1 transposon insertions in genes essential for phage infection were enriched, recovered and sequenced. Reads mapping statistics are provided in Table S3. Resampling analyses identified 36 genes enriched in the presence of Φ*Xhv*‐1 (Table 1). Functional analysis with COG categories revealed that most of these genes were associated with membrane biogenesis, particularly LPS and polysaccharide biosynthesis. Three genes were annotated as hypothetical or associated with putative functions. The main gene clusters involved in LPS biosynthesis in *X. hortorum* pv. *vitians* are illustrated in Figure 3.

**TABLE 1:** List of the 36 genes involved in Φ*Xhv*‐1 infection identified by Tn‐seq.

Locus	Gene	Product	nTA^a^	Log_2_FC^b^	*p*	*q*	COG classification
XHV734_2249	*lapB*	Lipopolysaccharide assembly protein B	17	5.06	0	0	Carbohydrate transport and metabolism (G)
XHV734_2250	—	UDP‐*N*‐acetylmuramyl pentapeptide phosphotransferase/UDP‐N‐acetylglucosamine‐1‐phosphate transferase	11	5.47	0	0	Cell wall/membrane/envelope biogenesis (M)
XHV734_2251	—	Epimerase/dehydratase protein	25	5.45	0	0	Carbohydrate transport and metabolism (G) Cell wall/membrane/envelope biogenesis (M)
XHV734_2252	*galU*	UTP‐glucose‐1‐phosphate uridylyltransferase	9	5.18	0	0	Cell wall/membrane/envelope biogenesis (M)
XHV734_3113	*udg*	UDP‐glucose 6‐dehydrogenase	19	5.2	0	0	Cell wall/membrane/envelope biogenesis (M)
XHV734_3482	—	Glycosyltransferase	27	5.7	0	0	Cell wall/membrane/envelope biogenesis (M)
XHV734_3756	*kdkA*	3‐deoxy‐d‐manno‐octulosonic acid kinase	5	5.32	0	0	Nucleotide transport and metabolism (F)
XHV734_3757	—	ADP‐heptose‐LPS heptosyltransferase	10	7.66	0	0	Cell wall/membrane/envelope biogenesis (M)
XHV734_3758	—	Hypothetical protein	5	7.61	0.0003	0.00063	Cell wall/membrane/envelope biogenesis (M)
XHV734_4092	*ahcY*	Adenosylhomocysteinase	11	2.46	0.0003	0.00063	Coenzyme transport and metabolism (H)
XHV734_4166	—	Glycosyl transferase	3	8.75	0.0018	0.00331	Function unknown (S)
XHV734_4222	—	Hypothetical protein	29	9.16	0	0	Cell wall/membrane/envelope biogenesis (M)
XHV734_4228	*cpsG*	Phosphomannomutase	17	9	0	0	Carbohydrate transport and metabolism (G)
XHV734_4229	*cpsB*	Mannose‐1‐phosphate guanyltransferase	16	7.68	0	0	Carbohydrate transport and metabolism (G) Cell wall/membrane/envelope biogenesis (M)
XHV734_4230	*rmlD*	dTDP‐4‐dehydrorhamnose reductase subunit, NAD(P)‐binding, of dTDP‐L‐rhamnose synthase	14	8.82	0	0	Cell wall/membrane/envelope biogenesis (M)
XHV734_4231	*rmlC*	dTDP‐4‐deoxyrhamnose‐3,5‐epimerase	9	9.06	0	0	Cell wall/membrane/envelope biogenesis (M)
XHV734_4232	*rmlA*	Glucose‐1‐phosphate thymidylyltransferase	18	9.06	0	0	Cell wall/membrane/envelope biogenesis (M)
XHV734_4233	*rmlB*	dTDP‐glucose 4,6‐dehydratase	17	9.06	0	0	Cell wall/membrane/envelope biogenesis (M)
XHV734_4236	*wxcK*	dTDP‐3‐amino‐3,6‐dideoxy‐alpha‐D‐galactopyranose transaminase	20	9.39	0	0	Amino acid transport and metabolism (E)
XHV734_4237	*wxcL*	Glycosyltransferase	21	9.32	0	0	Cell wall/membrane/envelope biogenesis (M)
XHV734_4238	*wxcM*	Bifunctional acetyl transferase/isomerase	15	9.52	0	0	Function unknown (S)
XHV734_4239	*wxcN*	Sugar transferase	12	9.06	0	0	Function unknown (S)
XHV734_4240	*wxcO*	Sugar translocase	28	9.19	0	0	Cell wall/membrane/envelope biogenesis (M)
XHV734_4241	*rmd*	UDP‐glucose 4‐epimerase	12	9.23	0	0	Carbohydrate transport and metabolism (G) Cell wall/membrane/envelope biogenesis (M)
XHV734_4242	*gmd*	GDP‐d‐mannose dehydratase, NAD(P)‐binding	17	9.27	0	0	Cell wall/membrane/envelope biogenesis (M)
XHV734_4243	*wxcE*	Glycosyltransferase	10	9.19	0	0	Not in COG
XHV734_4244	*wxcD*	Glycosyltransferase	33	9.2	0	0	Cell wall/membrane/envelope biogenesis (M)
XHV734_4245	*wxcC*	Glycosyl transferase	26	9.19	0	0	Cell wall/membrane/envelope biogenesis (M)
XHV734_4246	*wxcB*	Kinase	41	9.14	0	0	Coenzyme transport and metabolism (H) Transcription (K) Signal transduction mechanisms (T) Replication, recombination and repair (L)
XHV734_4247	*wzt*	ATP binding component of ABC‐transporter	34	9.21	0	0	Carbohydrate transport and metabolism (G) Cell wall/membrane/envelope biogenesis (M)
XHV734_4248	*wzm*	Transport permease protein	15	9.24	0	0	Intracellular trafficking, secretion, vesicular transport (U)
XHV734_4249	*wxcA*	Glycosyl transferase	36	9.06	0	0	Cell wall/membrane/envelope biogenesis (M)
XHV734_4250	*metC*	Cystathionine beta‐lyase	8	9.24	0	0	Amino acid transport and metabolism (E)
XHV734_4495	—	Putative CDP‐glycerol glycerophosphotransferase	12	9.12	0	0	Cell wall/membrane/envelope biogenesis (M)
XHV734_4496	—	Lipopolysaccharide core biosynthesis glycosyl transferase	16	9.14	0	0	Function unknown (S)
XHV734_4497	—	Lipid A core‐O‐antigen ligase‐like enyme	15	9.18	0	0	Cell wall/membrane/envelope biogenesis (M)

^a^ Number of TA sites.

^b^ Log_2_ fold change.

The involvement of these genes in phage resistance to Φ*Xhv*‐1 was confirmed by constructing LPS gene region deletion mutants (i.e., AB16734 ΔLPS2 and AB16734 ΔLPS3) and by identifying four transposition mutants from the Tn‐seq screen: AB16734 *wxcK* ^−^, AB16734 *wxcO* ^−^, AB16734 *wxcA* ^−^, AB16734 *cpsG* ^−^. All six mutants displayed complete resistance to Φ*Xhv*‐1 in both spotting assays (no lysis at 10^8^ PFU) and growth monitoring in liquid culture. Their growth kinetics were similar to the wild‐type strain in the absence of phage (Figure S2A). Using SYBR Gold‐labelled Φ*Xhv*‐1, phage attachment and DNA injection were monitored at the single‐cell level. All wild‐type LM16734 showed strong green fluorescence under blue light, confirming efficient adsorption and infection. In contrast, none of the AB16734 ΔLPS2, AB16734 ΔLPS3, AB16734 *wxcK* ^−^, AB16734 *wxcA* ^−^ or AB16734 *cpsG* ^−^ mutants emitted any fluorescence, and only one AB16734 *wxcO* ^−^ mutant cell showed fluorescence. These results demonstrate that phage adsorption, and thus infection, is either completely abolished or severely impaired in these mutants, validating the Tn‐seq findings (Figure S2B).

### 3.4 Phage Resistance Trade‐Offs With Virulence in *X. hortorum* pv. *vitians*

**FIGURE 4:**
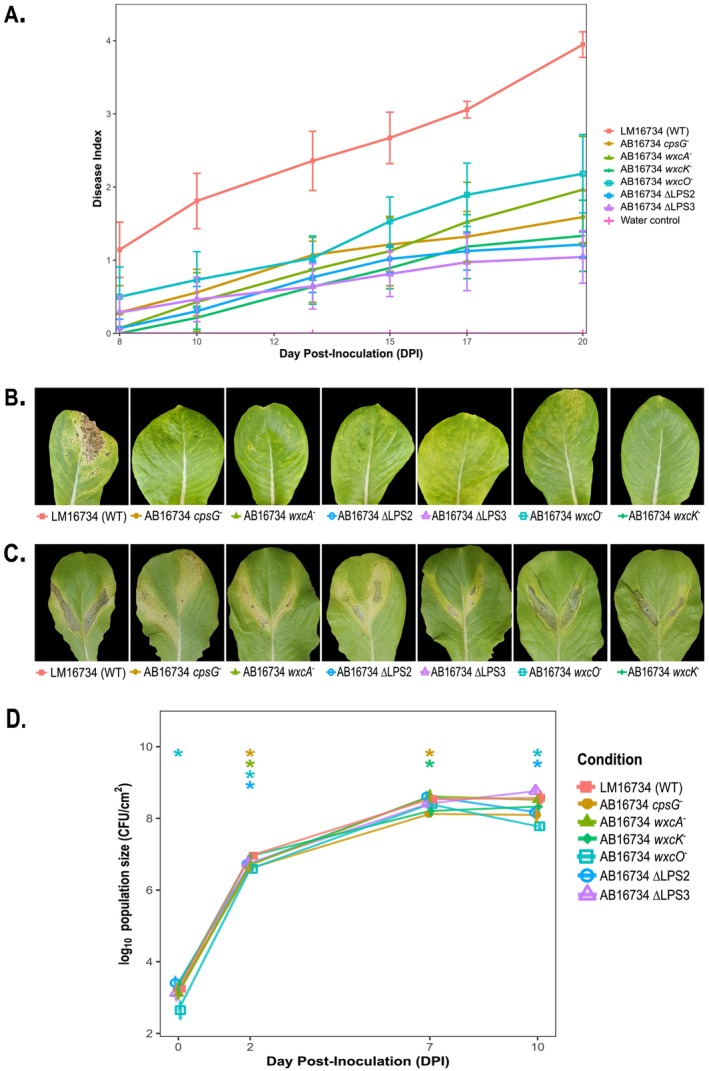
Trade‐off between phage resistance and bacterial pathogenicity in planta. (A) Dynamics of disease development over time (days post‐inoculation, DPI) for the wild‐type strain (LM16734) and phage‐resistant mutants. Data represent mean ± standard deviation (SD) from three independent replicates per condition. Statistical analysis using the Kruskal–Wallis test revealed significant differences among strains (*p* = 1.18 × 10^−32^). Pairwise Wilcoxon tests with Bonferroni correction indicated significantly reduced pathogenicity in all mutants compared to the wild‐type (*p*_adj_ < 0.05) at all measured DPI. (B, C) Representative disease symptoms observed on lettuce leaves at DPI 13 and 10, respectively. (B) Spray inoculation assay: symptoms following bacterial spray inoculation, simulating natural infection through stomatal entry. (C) Infiltration assay: symptoms observed after direct bacterial infiltration into the leaf apoplast, bypassing natural entry barriers to assess bacterial fitness within plant tissues. (D) Bacterial population dynamics of Φ*Xhv*‐1 resistant mutants following leaf tissue infiltration. In planta fitness of wild‐type *X. hortorum* pv. *vitians* LM16734 and Φ*Xhv*‐1 resistant mutants was assessed after direct infiltration into lettuce leaves. Evolution of bacterial population size was quantified at 0, 2, 7, and 10 DPI and expressed as log_10_ CFU/cm^2^ of inoculated leaf. At each time point, bacteria were recovered from surface‐sterilised leaf tissues and quantified by serial dilution and plating. Each point represents the mean bacterial concentration from three independent biological replicates, with error bars indicating the 95% confidence interval (CI). Statistical differences between each mutant and the wild‐type strain at each time point were assessed using Wilcoxon rank‐sum tests, followed by Bonferroni correction for multiple comparisons. Significant differences are denoted by asterisks (*p < 0.05, **p < 0.01, ***p < 0.001).

**FIGURE 5:**
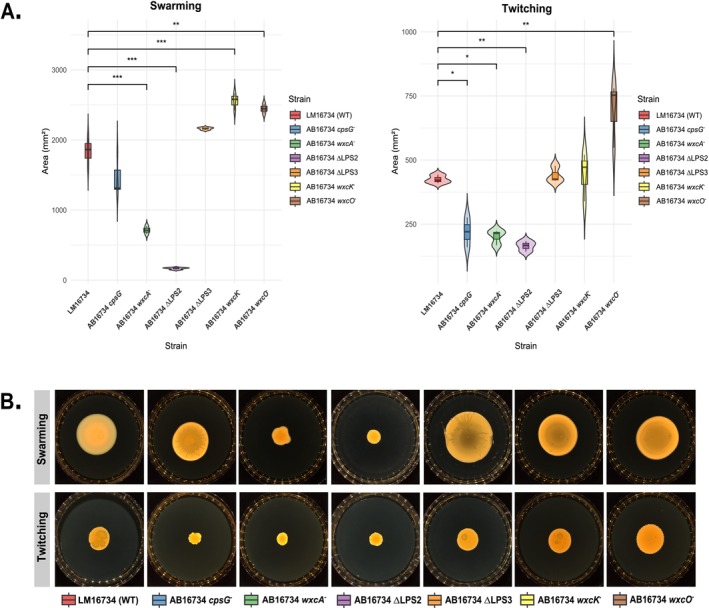
Pleiotropic effects of phage resistance mutations on bacterial motility, a key determinant of virulence. (A) Swarming and twitching motility assays for the wild‐type strain (LM16734) and phage‐resistant mutants. Violin and boxplots represent the distribution of motility zone areas (mm^2^) for each strain. Data represent three independent replicates per condition. Statistical analysis using ANOVA, followed by Dunnett's post hoc test with Benjamini–Hochberg correction, revealed significant motility differences between the wild‐type and several mutants (*p*_adj_ < 0.05). Asterisks indicate statistical significance (*p < 0.05, **p < 0.01, ***p < 0.001). (B) Representative images of swarming (top row) and twitching (bottom row) motility assays.

Among the 36 genes identified as critical for Φ*Xhv*‐1 infection, 23 overlapped with genes previously shown to contribute to bacterial fitness in planta (Morinière et al. 2022), including those located in the *cps*‐*rml* regions, LPS Regions 1 and 2 genes cluster (Figure 3). Although mutations in the LPS3 gene cluster showed the highest fitness gains in the Tn‐seq experiment when exposed to the phage, the previous in planta screen suggested they were not essential for host–plant interaction. To assess whether mutations conferring phage resistance impacted pathogenicity, symptom development was monitored in lettuce plants. All resistant mutants caused delayed symptom onset and significantly reduced disease severity, with approximately 50% less symptom development observed 20 DPI compared to the wild‐type strain (Figure 4A,B). To explore possible causes of this reduced virulence, bacterial multiplication was quantified following direct infiltration into leaf tissues. All strains reached similar population levels (~10^8^ CFU cm^−2^) after 10 days, with only minor reductions observed for mutants AB16734 ΔLPS2 and AB16734 *wxcO* ^−^ as determined by Wilcoxon tests with Bonferroni correction (Figure 4D). Despite comparable bacterial densities, tissues infiltrated with mutant strains consistently showed reduced symptom expression (Figure 4C). Since bacterial motility is essential for plant surface colonisation and entry, swarming and twitching capacities of the mutants were next evaluated. Swarming was significantly reduced in ΔLPS2 knockout mutant and the AB16734 *wxcA* ^−^ transposon mutant, with reductions of 91% and 61%, respectively, compared to the wild type (Figure 5). Twitching was also impaired, with colony diameter reductions of 48%, 52% and 61% observed for the AB16734 *cpsG* ^−^, AB16734 *wxcA* ^−^ and AB16734 ΔLPS2 mutants, respectively (Figure 5). In contrast, transposon mutants in the LPS3 region exhibited a significant increase in swarming motility, with a 39% and 33% enhancement for AB16734 *wxcK* ^−^ and AB16734 *wxcO* ^−^, respectively, as well as a 63% increase in twitching motility for *wxcO* ^*−*^.

## 4 Discussion

Phage‐based biocontrol represents a promising strategy for the management of bacterial plant diseases. However, its long‐term efficacy requires a comprehensive understanding of phage–host interactions and the evolutionary consequences of resistance. In this context, we isolated and characterised the newly lytic phage Φ*Xhv*‐1 and identified its bacterial receptors in *X. hortorum* pv. *vitians*. Using a combination of genomic analyses, high‐throughput genetic screening (Tn‐seq), targeted mutagenesis and phenotypic analyses, we elucidated key molecular determinants of phage susceptibility and their trade‐off for virulence in planta.

Φ*Xhv*‐1 displays several required genomic features for biocontrol, notably the absence of lysogeny‐related genes, integrases and elements associated with virulence and antibiotic resistance (Fernández et al. 2019). In addition, its narrow host range, limited to the pathovar *vitians*, demonstrates its specificity for this pathogen alone. Other phages specific to *X. hortorum* pv. *vitians* will have to be combined with Φ*Xhv*‐1 to form a cocktail targeting the full diversity of this pathogen while avoiding off‐target effects. These complementary phages could be sourced from environmental sampling or experimental evolution for host range expansion (Burrowes et al. 2019; Mapes et al. 2016). A better understanding of host range determinants would help optimise phage selection for the formulation of effective cocktails. A recent large‐scale study in *E. coli* suggested that, despite the influence of intracellular anti‐phage defence systems, surface receptor polymorphisms have a predominant impact on susceptibility (Gaborieau et al. 2024). Thus, it is crucial to precisely identify these bacterial receptors to inform effective biocontrol strategies.

To investigate the molecular basis of the interaction between Φ*Xhv*‐1 and *X. hortorum* pv. *vitians* LM16734, a Tn‐seq screen was performed, identifying 36 genes required for phage susceptibility. Consistent with other Tn‐seq studies, most identified mutations impacted genes encoding bacterial surface receptors (Holtappels et al. 2022; Mutalik et al. 2020; Adler et al. 2021; Kortright et al. 2020). Notably, 19 of these genes form a contiguous 26 kb cluster region previously identified as the LPS biosynthesis cluster in *X. hortorum* pv. *vitians* (Morinière et al. 2022). This cluster, conserved and previously characterised by mutagenesis in *X. campestris* pv. *campestris* (Vorhölter et al. 2001; Steffens et al. 2016), comprises five functional regions: *cps* (or *xan*), *rml*, LPS, LPS2 and LPS3. These regions are involved in the synthesis of nucleotide sugar precursors (e.g., rhamnose), the biosynthesis of the rhamnose‐based O antigen (OAg) backbone, and its side chain modifications (Vorhölter et al. 2001; Molinaro et al. 2002). Functional insights into these genes, inferred from studies in *X. campestris* pv. *campestris*, highlight their critical roles in nucleotide sugars metabolic pathways required for LPS and xanthan biosynthesis (Steffens et al. 2016). For instance, *cpsG*, acting upstream in the pathway, contributes to the synthesis of GDP‐D‐mannose, GDP‐D‐rhamnose and UDP‐D‐glucose, key components of both xanthan and LPS core (Vorhölter et al. 2001; Köplin et al. 1992). Its disruption would likely result in the loss of both the oligosaccharide core and OAg (Vorhölter et al. 2001). Genes within the LPS1 region direct the assembly of rhamnose‐based OAg subunits (Steffens et al. 2016; Molinaro et al. 2002); *wxcA*, for example, encodes a glycosyltransferase whose inactivation eliminates the OAg without altering other LPS components (Vorhölter et al. 2001). Consistent with these data, the *wxcA* mutation in *X. hortorum* pv. *vitians* results in smaller, matte and less mucoid colonies, consistent with a rough LPS phenotype. Among the identified genes, those in the LPS3 region showed the highest selective value score in the Tn‐seq data and their disruption conferred complete resistance to Φ*Xhv*‐1 without impacting in vitro growth. This region is predicted to encode enzymes involved in the synthesis and transfer of N‐acetyl‐fucosamine (Fuc3Nac) residues, which form side branches on the OAg. In *X. campestris* pv. *campestris*, a mutant in *wxcK* exhibits elongation of the rhamnose backbone and loss of side chains (Steffens et al. 2016). Further biochemical analyses are needed to confirm similar structural alterations occur in *X. hortorum* pv. *vitians*.

**FIGURE 6:**
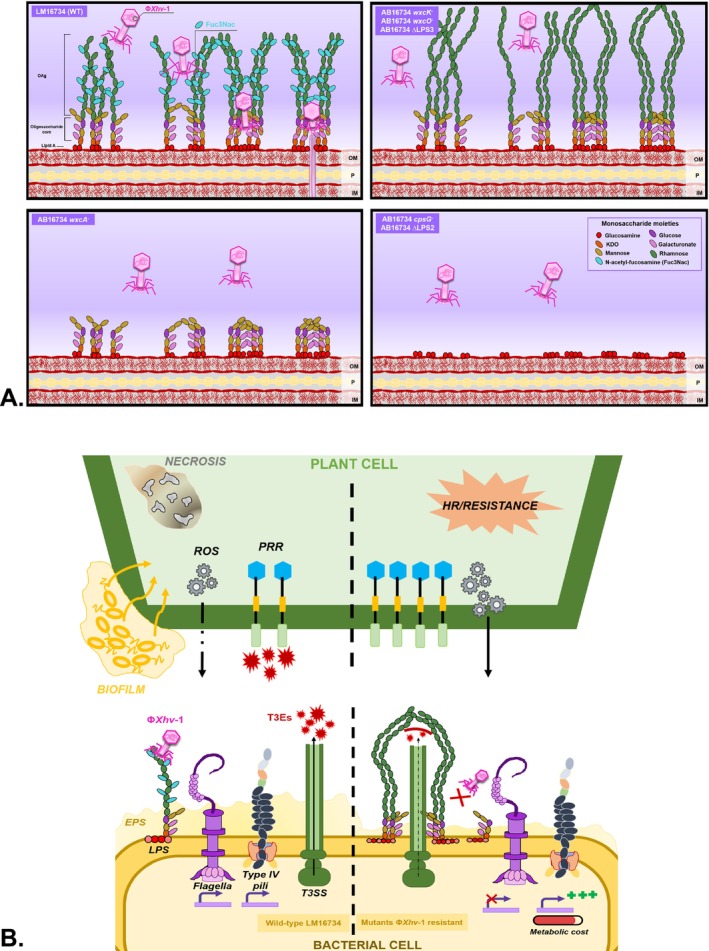
LPS as a dual determinant of phage sensitivity and bacterial virulence in *X. hortorum* pv. *vitians*. (A) Schematic representation of the proposed adsorption process of phage Φ*Xhv*‐1 on *X. hortorum* pv. *vitians* LM16734 (WT) and its phage‐resistant LPS mutants, based on sensitivity profiles and genetic inferences from *X. campestris* pv. *campestris*. Φ*Xhv*‐1 recognises specific *N*‐acetyl‐fucosamine (Fuc3Nac) side chains within the O‐antigen (OAg) as a primary receptor. A secondary interaction with an unidentified component of the core‐oligosaccharide (CO‐OS) stabilises phage attachment and enables DNA injection. Structural alterations in the OAg structure (elongated of the rhamnose backbone without Fuc3Nac branches, or complete OAg absence) or loss of the entire LPS (OAg and CO‐OS) prevent phage adsorption. Monosaccharide moieties are detailed in (A). (B) Theoretical model of the pleiotropic effects associated with LPS mutations conferring Φ*Xhv*‐1 resistance. The vertical dashed line separates the wild‐type strain (left) from phage‐resistant mutants (right), illustrating both bacterial and host interaction outcomes. Wild‐type LM16734, with a complete LPS structure, supports efficient phage adsorption and full virulence, including T3SS effector delivery into the plant cell, significant EPS/biofilm production, and balanced surface motility. In contrast, LPS‐altered mutants exhibit impaired phage binding and multiple fitness trade‐offs. These include attenuated in planta virulence, which could be attributed to impaired T3SS functionality and/or potentially enhanced recognition of modified LPS by plant PRRs (triggering ROS production and hypersensitive response). Phage‐resistant mutants also display altered surface motility, including reduced motility which would impair their ability to colonise tissues. In some cases, hypermotility is observed, which may impose an additional metabolic cost and could potentially affect EPS/biofilm formation. This schematic is a theoretical model that integrates observations from this study with mechanistic hypotheses developed in the discussion. Not drawn from a single experimental result. IM, inner membrane; OM, outer membrane; P, peptidoglycan.

Our findings support a model for Φ*Xhv*‐1 attachment on LM16734. Tailed phage typically recognise two successive bacterial receptors (Nobrega et al. 2018). The absence of adsorption coupled with the resistance phenotype of LPS3‐region mutants lacking OAg side branches suggests that Φ*Xhv*‐1 primarily recognises Fuc3Nac side residues as its initial adsorption receptor. As porin‐encoding genes were absent from our Tn‐seq hits, the second receptor is likely a component of the LPS oligosaccharide core, or reversible adsorption to the OAg is a prerequisite for irreversible binding to an outer membrane protein (Figure 6A). The restricted host range of Φ*Xhv*‐1 might be explained by the known inter‐strain variations in LPS biosynthetic genes reported across several *Xanthomonas* species (Patil et al. 2007). Comparative genomic analyses of *X. campestris* pv. *campestris* strains have revealed significant divergence within the *wxc* gene cluster (Vorhölter et al. 2001; He et al. 2007). This variability has been identified as a critical determinant influencing the infectivity of phage *Foxunavirus* (Holtappels et al. 2022). Such potential polymorphisms within the *wxc* cluster of *X. hortorum* pv. *vitians* could lead to structural variations in its OAg, and consequently affect Φ*Xhv*‐1 infectivity, as observed for phages infecting *X. campestris* pv. *campestris*.

In addition to their involvement in phage interaction, gram‐negative bacterial LPS plays main roles in host–pathogen interactions. These molecules cover more than 90% of the bacterial cell surface and protect bacterial cells from environmental stresses, including oxidative bursts (Rosenfeld and Shai 2006; Raetz and Whitfield 2002; Petrocelli et al. 2012), and also facilitate evading the host immune system by camouflaging pathogens (Aslam et al. 2008; Silipo et al. 2010). Paradoxically, LPS is also a major microbial‐associated molecular patterns (MAMPs) recognised by host immune receptors. In plants, they trigger defence responses, such as the expression of pathogenesis‐related genes and the thickening of the cell wall structure (Dow et al. 2000; Newman et al. 2000; Matsuura 2013). Parts of the LPS (i.e., core and O‐antigen) are also major virulence factors for phytopathogenic bacteria such as *Xanthomonas*, *Pseudomonas* and *Ralstonia* (Büttner and Bonas 2010; Helmann et al. 2020; Su et al. 2021). Attenuated or abolished virulence has been demonstrated in OAg biosynthesis mutants of *X. campestris* pv. *campestris*, *Xanthomonas oryzae* pv. *oryzae* and *Xanthomonas alfalfae* subsp. *citrumelonis* (Qian et al. 2005; Wang et al. 2013; Vicente and Holub 2013). In a previous Tn‐seq experiment in planta, with the exception of the LPS3 region, the regions involved in the biosynthesis of LPS described above were identified as critical genetic factors for successful *X. hortorum* pv. *vitians* invasion and survival inside lettuce leaves (Morinière et al. 2022).

Given that this LPS biosynthesis cluster is also required for Φ*Xhv*‐1 infection, mutations conferring phage resistance are likely to compromise bacterial virulence and fitness on lettuce. This trade‐off, in which resistance to phage infection comes at the cost of reduced pathogenicity, was consistently observed across mutants in the *cps*, LPS1, LPS2 and LPS3 regions. These findings suggest that structural disruptions in LPS, particularly the loss or modification of the OAg, interfere with key virulence functions (Figure 6B). One possible mechanism involves the type III secretion system (T3SS), whose activity may be impaired by OAg elongation, potentially affecting the efficient delivery of type III effectors. Supporting this hypothesis, studies in *E. coli* and *Salmonella enterica* have shown that elongated OAg can hinder host cell entry and reduce T3SS functionality (Hölzer et al. 2009; Liu et al. 2021). Additionally, in plant hosts, such LPS modification may also enhance recognition by immune receptors, triggering a hypersensitive response (HR) and preventing successful colonisation. Alongside reduced virulence, several LPS‐region mutants exhibited altered surface motility, including defects in swarming and twitching. Since these forms of motility are essential for *Xanthomonas* to reach and colonise foliar tissues (Corral et al. 2020; Meng et al. 2011), such phenotypes likely contribute to the observed attenuation. Furthermore, type IV pili‐mediated twitching has been implicated in virulence and biofilm formation in other phytopathogenic bacteria, such as *Ralstonia solanacearum* (Bhuyan et al. 2024; Athinuwat et al. 2018; Felipe et al. 2018; Corral et al. 2020), reinforcing the relevance of these observations. Interestingly, certain LPS3 mutants displayed the opposite phenotype, with significantly increased swarming and twitching motility. Although surface motility is typically associated with virulence, this paradoxical association between hypermotility and attenuated virulence has also been observed in phytopathogenic models. In *Ralstonia solanacearum* , a hypermotile *motN* mutant displayed impaired virulence, attributed to reduced biofilm formation (Meng et al. 2011). In *X. oryzae* pv. *oryzae*, deletion of *phaR* similarly resulted in hypermotility but caused a decrease in exopolysaccharide (EPS) production and triggered basal plant immunity (Long et al. 2018). As EPS and biofilm protect pathogens from host defences and environmental stress, their reduction likely impairs infection. Moreover, excessive motility may carry a metabolic cost: in *E. coli* , approximately 2% of the total cellular energy is allocated to the synthesis, regulation, and rotation of flagella (Macnab 1996), potentially further limiting bacterial fitness in planta.

This trade‐off between phage resistance and virulence on lettuce observed in *X. hortorum* pv. *vitians* is consistent with findings in other pathosystems, both in plant‐pathogenic bacteria (Bartnik et al. 2022; Holtappels et al. 2020; Zhang et al. 2022; Brockhurst et al. 2021; Meaden et al. 2015) and human pathogens (Kortright et al. 2022; Sumrall et al. 2021; Olszak et al. 2019). Most studies have demonstrated this phenomenon using laboratory‐induced mutations generated during short‐term co‐culture with phages. For example, spontaneous mutants of *Dickeya solani* resistant to the φD5 phage, carrying mutations in the *hlyD* and *tuf* genes, were isolated in vitro and exhibited reduced fitness and virulence in planta (Sokolova et al. 2023). However, studies investigating the resistance of plant pathogenic bacteria to phages in natural environments following phage treatments remain scarce, and the results are contrasted compared to in vitro studies. The most compelling study to date presented the results of a coevolution experiment between *Pseudomonas syringae* pv. *tomato* and two lytic bacteriophages, showing that no phage‐resistant mutants emerged in natural environments (i.e., the tomato leaf apoplast), while resistance rapidly developed and reached high frequencies under in vitro conditions with rich media (Hernandez and Koskella 2019). Conversely, another study reported the isolation of a *Pseudomonas syringae* pv. *actinidiae* mutant resistant to phage ΦPsa374 in planta. In this case, the mutant harbouring a mutation in an LPS glycosyltransferase maintained full virulence (Warring et al. 2022). These conflicting results indicate the need for further investigation into phage–bacteria interactions, with a particular focus on the analysis of phage‐targeted receptors and their role in pathogenicity. They also underscore the importance of designing phage cocktails targeting diverse bacterial receptors as a foundation for sustainable and effective phage‐based biocontrol strategies (Holtappels et al. 2021).

## 5 Conclusion

Sustainable phage‐based biocontrol hinges on precise characterisation and a thorough understanding of bacterial resistance mechanisms. Here, we characterised the newly isolated phage Φ*Xhv*‐1, a member of a novel *Caudoviricetes* genus, highlighting key features relevant for biocontrol. Despite its effectiveness on strains reflecting the diversity of *X. hortorum* pv. *vitians*, the phage Φ*Xhv*‐1 will need to be combined with other well‐characterised phages in order to cover the full diversity of this pathogen. Our study leveraged Tn‐seq and targeted mutagenesis to demonstrate the critical role of LPS in Φ*Xhv*‐1 adsorption. Elucidating such phage–bacteria interactions for other active phages will enable the rational design of durable treatment by selecting phages with diverse host molecular targets. Crucially, the significant reduction in bacterial virulence observed with LPS mutations that confer Φ*Xhv*‐1 resistance suggests a powerful fitness trade‐off. These findings indicate that, even if phage resistance emerges in field conditions, the associated fitness cost would likely restrict the ecological success and pathogenic potential of resistant mutants. Consequently, phage‐based biocontrol may remain effective, either by eliminating susceptible bacteria or by selecting attenuated, less virulent strains. This study significantly advances phage‐based control strategies against *X. hortorum* pv. *vitians* and deepens our understanding of *Xanthomonas* phage receptors.

## Author Contributions

**Anaelle Baud:** conceptualization, investigation, writing – original draft, methodology, visualization, formal analysis, data curation. **Lucas Morinière:** formal analysis, software, writing – review and editing. **Imane El Idrissi:** writing – review and editing, investigation, formal analysis. **Fernando Clavijo‐Coppens:** investigation, writing – review and editing, visualization. **Elise Lacroix:** investigation, writing – review and editing, methodology. **Nicolas Taveau:** investigation, visualization, writing – review and editing. **Denis Costechareyre:** writing – review and editing, funding acquisition, validation, project administration, resources. **Franck Bertolla:** writing – review and editing, project administration, funding acquisition, conceptualization, supervision, resources, validation, methodology, investigation.

## Conflicts of Interest

The authors declare no conflicts of interest.

## Supporting information


**Data S1:** Supplementary methods.

**Figure S1:** Plaque morphology of Φ*Xhv*‐1 on a 0.6% soft agar TSB plate seeded with *X. hortorum* pv. *vitians* LM16734, forming circular and clear plaques.

**Figure S2:** Phage Φ*Xhv*‐1 resistance phenotype in mutants revealed by growth kinetics and fluorescence‐based adsorption dynamics. (A) Growth kinetics of wild‐type *X. hortorum* pv. *vitians* LM16734 and mutants in liquid culture, with and without Φ*Xhv*‐1 infection. Optical density at 600 nm (OD_600_) was recorded every 20 min over 25 h. Each point represents the mean absorbance from five technical replicates, with error bars indicating the 95% confidence interval. While the wild‐type strain (black curve) showed significant growth inhibition, mutants exhibited sustained growth, confirming their resistance to Φ*Xhv*‐1. (B) Fluorescence microscopy analysis of phage adsorption to bacterial cells. Wild‐type and transposon mutants were infected with Φ*Xhv*‐1 at a multiplicity of infection (MOI) of 400 using SYBR Gold‐stained Φ*Xhv*‐1 particles. After incubation and washing, images were acquired under natural light (left) and fluorescence (right) conditions (*λ*Ex = 495 nm, *λ*Em = 537 nm). Strong fluorescence in the wild‐type strain indicates efficient phage adsorption, while mutants exhibited significantly reduced fluorescence, suggesting impaired phage binding or entry.

**Table S1:** List of bacterial strains and plasmids used or generated in this study.
**Table S2:** List of primers used in this study. Sequences corresponding to restriction sites are underlined and sequences non‐homologous to *Xhv* are represented in lowercase and italics.
**Table S3:** Transposon‐insertion sequencing (Tn‐seq) statistics before Totreads normalisation.
**Table S4:** Predicted coding sequences and functional annotations of the ΦXhv‐1 genome.

## Data Availability

The assembled and annotated genome of the phage Φ*Xhv*‐1 has been deposited in GenBank under accession number PV408261. The NCBI GenBank assembly accession number for the complete *X. hortorum* pv. *vitians* LM16734 genome is GCA_014338485.1. Raw sequencing reads for the phage Φ*Xhv*‐1 genome have been deposited in the NCBI SRA database under BioProject PRJNA1277312, accession SRP596415. Transposon insertion sequencing raw reads have been deposited in the NCBI SRA database under accession numbers SRR32994741 to SRR32994744 and are associated with BioProject no. PRJNA1247032. In addition, raw experimental data from wet lab assays have been made publicly available via Zenodo: https://doi.org/10.5281/zenodo.15678039.
