# Supplementary material for: Unveiling Host Interactions and Evolutionary Constraints of a Novel Bacteriophage Infecting Xanthomonas hortorum pv. vitians
Source: Environ Microbiol Rep. 2025 Oct 30;17(6):e70171. doi: 10.1111/1758-2229.70171 (PMC12573098; doi:10.1111/1758-2229.70171)
Supplement: Supplementary file 1 — Data S1: Supplementary methods. [file EMI4-17-e70171-s002.docx]

**Unveiling host interactions and evolutionary constraints of a novel bacteriophage infecting *Xanthomonas hortorum* pv. *vitians***

**Anaelle Baud^1,*^, Lucas Morinière^1^, Imane EL Idrissi^1^, Fernando Clavijo-Coppens^2^, Elise Lacroix^1^, Nicolas Taveau^2^, Denis Costechareyre^2^, Franck Bertolla^1,*^**

**Author affiliations**:

^1^ Universite Claude Bernard Lyon 1, Laboratoire d'Ecologie Microbienne, UMR CNRS 5557, UMR INRAE 1418, VetAgro Sup, 69622 Villeurbanne, France.

^2^ GREENPHAGE, 34830 Clapiers, France.

***Correspondence:** Franck Bertolla, franck.bertolla@univ-lyon1.fr; Anaelle Baud, anaelle.baud@univ-lyon1.fr

**Supplementary method**

**Bacterial strains, plasmids and growth conditions**. The bacterial strains and plasmids used in this study are listed in **Table S1**. *Xanthomonas hortorum* pv. *vitians* strains were routinely cultured on tryptic soy agar (TSA) and in tryptic soy broth (TSB) at 28°C. For all experiments with phages, media were supplemented with 1 or 10 mM CaCl_2_ to promote adsorption. *Escherichia coli* strains were grown on Lysogeny Broth (LB) medium at 37°C. The strains were kept at -80°C in 30% (v/v) glycerol vials for long-term storage. When required, the media were supplemented with 100 µg.mL^-1^ ampicillin, 25 µg.mL^-1^ kanamycin, 30 µg.mL^-1^ chloramphenicol, and 50 µg.mL^-1^ for cycloheximide. Sucrose was added at 5% (w/v) final concentration.

**Isolation and amplification of the phage.** Wastewater samples from treatment plants in Montpellier (France) were used to phage isolation. Effluent samples were centrifuged at 5,000 x g for 10 min at room temperature, and the supernatants were filtered through a 0.2 μm filter to remove debris. Three milliliters of filtered effluent were mixed with 7 mL of *X. hortorum* pv*. vitians* culture in early logarithmic phase (OD_600_ ~ 0.5). After overnight incubation (150 rpm, 28 °C), cultures were centrifuged at 5,000 x g for 10 min, and the resulting supernatants were filtered through a 0.2 µm filter. Phages were isolated using the double-layer agar technique (1). Briefly, 100 µL of enriched samples were mixed with 500 µL of *X. hortorum* pv*. vitians* overnight culture and 4 mL of melted soft TSA (0.6 % agar) overlay, and then poured on a TSA plate. After incubation for 18 h at 28 °C, single clear plaques were picked by micropipette aspiration using filter tips and resuspended in 2.5 mL of SM buffer (50 mM Tris-HCl [pH 7.5], 8 mM MgSO_4_.7H_2_O, 100 mM NaCl). The mixture was stirred at 4°C for 1 h and filtered on 0.2 µm. A single plaque was collected two subsequent times to generate pure phage isolates. Concentrated phage stocks were kept at 4°C in SM buffer.

**Phage morphological characterization by transmission electronic microscopy (TEM)**. Phage particles were sedimented via centrifugation (21,800 × g, 90 min, 4 °C), and the pellet was further washed in 0.1 M acetate ammonium buffer by repeating twice the centrifugation step. Subsequently, phage suspensions were dried on a 300-mesh grid coated with formvar (Delta Microscopies) for 2 min at room temperature, and excess solution was removed using filter paper. The grids were negatively stained with 2% uranyl acetate. Grids were washed with two 10µL-droplets of uranyl acetate 2 % and then stained by incubation with one 10µL-droplet of uranyl acetate 2 % during 30 s. Excess of uranyl acetate was drained on a blotting paper and grids were dried for 10 min before image acquisition. The grids were imaged via TEM using a JEOL 1400 Flash microscope operated at 120 kV.

**Whole genome sequencing of bacterial and phage genomes, comparative genomics**. Bacterial DNA were extracted with the Microbial DNA kit (Macherey-Nagel, Düren, Germany) following manufacturer’s instructions. All genomes were sequenced in Illumina HiSeq 2 x 150 bp at Novogene (Cambridge, United Kingdom). Paired-end reads were assembled in contigs using UNICYCLER v.0.5.0 with a minimum contig size of 200 bp and then annotated with the NCBI RefSeq pipeline. For phylogenomic analysis, a tree was inferred using the Genome BLAST Distance Phylogeny (GBDP) method via the online Type Strain Genome Server (TYGS) (2). FastME 2.1.4. was used for tree reconstruction, with a BioNJ starting tree and subtree purring and regrafting (SPR) post processing. Branch support values were calculated from 100 bootstrap replicates. Antiviral defense systems encode by bacteria were detected with the online tool DefenseFinder (3).

Phage DNA was purified according to Gendre *et al*., 2022 (4) without addition of SDS and proteinase K, and submitted to MiSeq Illumina sequencing 2 x 150 bp. Reads quality was evaluated with FastQC 0.11.90 (5) followed by a quality filtering by using PRINSEQ 0.20.4 (6). Reads were assembled using SPAdes 3.15.2 (7). Genome circularization was performed using UGENE 38.0 (8). Contigs were submitted to the Nucleotide database (https://www.ncbi.nlm.nih.gov/nucleotide/, 2019) of the BLAST software (9) to validate the purity of sequences. Assembly quality was evaluated with Bowtie2 2.4.4 (10) and Samtools 2.12 (11). Circular permutations were searched using the MAUVE software 2015-02-25 (12).BACPHLIP was used to predict the lifestyle of the phage Φ*Xhv*-1 (13).

**Adsorption assay.** Early-log-phase culture of the production strain (OD_600_ ≈ 0.2, 10^8^ CFU.mL^-1^) in 30 mL TSB supplemented with 10 mM CaCl_2_ was mixed with phages at a MOI of 10^-2^ (10^6^ PFU/mL). The co-culture was incubated at 28°C. Aliquots (1 mL) were collected every 10 min for 1 h. Samples were filtered to collect non-adsorbed (free) phages. Five-microliters drops of serial 10-fold dilutions were spotted onto soft TSB agar overlays containing the production strain to quantify unadsorbed phages, after overnight incubation at 28°C. Two independent experiments were performed, each consisting of three technical replicates for phage titration. At each time point, the percentage of free phages was calculated as the titer at that time relative to initial titer.

**One-step growth curves.** To assess the infectivity and replication dynamics of Φ*Xhv*-1, an early log-phase culture of the production strain was mixed with the phage at a MOI of 10^-1^. Co-culture was incubated at 28°C for the previously determined adsorption time. Aliquots were collected before and after the adsorption step. Non-adsorbed phages were removed by centrifugation (5,000 x *g*, 21°C, 10 min), and the cell pellet was washed twice before being resuspended in 35 mL of TSB supplemented with 10mM CaCl_2_. The resuspended co-cultures were incubated at 28°C with shaking at 160 rpm. Aliquots were collected over a period of 2 h. Samples were filtered to isolate free phage particles. Phage titers at each time point were determined using the spot assay as described above. The burst size was calculated as the ratio of the number of phages released after the rise period (corrected by subtracting the phage titer at t_0_) to the number of adsorbed phages. The number of adsorbed phages was determined by subtracting the titer of non-adsorbed phages remaining after the adsorption step from the initial phage input. Two independent experiments were performed, each with three technical replicates per time point.

**Bacterial growth inhibition assay.** Bacterial cultures were grown in TSB overnight and the OD_600_ were adjusted to 0.1 in fresh TSB and then placed under agitation at 160 rpm, 28°C, for 1 h. For each strain, 170 µL of the early exponential phase culture were inoculated with 8.5 µL of Φ*Xhv*-1 at 10^9^ PFU.mL^-1^ (MOI = 1). The well-plate was incubated for 30 min at room temperature to allow phage adsorption. Control cultures without the phage were also performed for each strain. OD_600_ were measured in 100-well Honeycomb plates with a Bioscreen C MBR BACTERIO (Thermo Fisher Scientific) every 20 min during 25 h. All experiments were done in three biological replicates, each consisting of 5 technical replicates.

**Phage host range.** The susceptibility of a collection of *X*. *hortorum* pv. *vitians* strains to Φ*Xhv*-1 was evaluated by growth monitoring as described above and by spot assays on double-layer agar plates. Briefly, 500 µL of an overnight culture of the tested strain (OD_600_ ~ 1) were mixed with 4 mL of soft TSB agar (0.6 %) supplemented with 1 mM CaCl_2_ and poured on TSA plates. Ten µL drops of serial dilutions of a concentrated suspension of Φ*Xhv*-1 at 10^10^ PFU.mL^-1^ were spotted onto bacterial lawns, followed by overnight incubation at 28°C. Strains were considered resistant when the efficiency of plating (EOP), calculated as the ratio of the phage titer on the tested strain to the titer on isolation strain, was ≤ 10^-4^. All experiments were performed in three biological replicates with two technical replicates. To assess the correlation between bacterial genetic relatedness and Φ*Xhv*-1 susceptibility, the phylogenetic signal of the sensitive/resistance phenotype (based on EOP values) was evaluated using the delta statistic (δ) with a thousand bootstrap replicates as described by Borges *et al*. (14).

**Genome-wide phage-resistance genes screen using transposon insertion sequencing**. The procedure for generating the Tn-seq mutant library was described previously (15). Transposon library aliquots were thawed at 4°C overnight and inoculated in flasks containing 30 mL of TSB supplemented with 10 mM CaCl_2_ to obtain an OD_600_ ~ 0.1 (equivalent to 5.10^7^ CFU/mL). Cultures were incubated during 1 h at 28 °C under shaking at 150 rpm until they reached an OD_600_ ~ 0.2. Then, Φ*Xhv*-1 was added to obtain a MOI ~ 1. Phage adsorption was promoted by incubating the cocultures without shaking for 30 min at 28°C, then shaking was resumed at 150 rpm during 21 h. Library grown without phage served as a control. Each condition was performed in duplicates. At the end, 25 mL of each cell suspensions were centrifuged at 5,000 x g for 10 min at 4°C and cell pellets were temporarily conserved at – 20°C. Genomic DNA library extraction and preparation were conducted as described previously (15) and were sent to the I2BC-sequencing platform (I2BC, Gif-sur-Yvette, France) to be sequenced in single-read 75-bp on a NextSeq 5000 instrument (Illumina, Inc.).

Before centrifugation, 1 mL-aliquots were collected, diluted and spread on TSA supplemented with kanamycin to isolate phage-resistant mutants. After two days of incubation at 28°C, the isolated mutants were stored in three 96-well plates in 30% (w/v) glycerol. A subset was identified by AP-PCR (16) and used for further analyses.

**Fitness assessment of genes associated with phage infection**. Sequencing reads pre-processing for further TnSeq analysis was performed as described previously (15). Read counts were normalized using the “Totreads” method available in TRANSIT v.3.2.1 (17) to obtain the same total number of reads for each sample. The contribution of each genetic feature to bacterial fitness was determined by performing pairwise comparisons between the phage condition and the control condition with the « Resampling » method available in TRANSIT v.3.2.1. Reads in the 5% N-terminal and 10% C-terminal portions of the genetic feature were discarded, and a LOESS (locally estimated scatterplot smoothing) correction for genome positional bias was applied. Genetic features with a log_2_ Fold Change (log_2_FC) > 2 and a q-value ≤ 0.05 were considered critical for successful phage infection of *X. hortorum* pv*. vitians* LM16734 by Φ*Xhv*-1.

**Construction of *X*. *hortorum* pv. *vitians* deletion mutants.** All the primer pairs used in this study are listed in **Table S2**. Knockout mutants of regions 2 and 3 of the LPS gene cluster were constructed using the *sacB* counter-selection system as described previously (18). Upstream and downstream 650-bp regions were amplified using specific primers and cloned into the multiple cloning site (MCS) of the pK18*mobsacB* plasmid using the T5-Exonuclease-Dependant-Assembly (TEDA) method (19). All constructions were verified by Sanger sequencing. Plasmids were introduced into *X. hortorum* pv. *vitians* LM16734 by triparental mating with the helper strain *E. coli* RK600 (20). In addition, genomes of the deletion mutants were sequenced.

**Phage adsorption assay by fluorescence microscopy.** Phage adsorption was evaluated for all resistant mutants and the wild type strain LM16734 using fluorescence microscopy as described previously (21) with some modifications. Briefly, 1 µL of a SYBR gold 1X solution was mixed with 10 mL of a phage suspension at 3.10^10^ PFU.mL^-1^. The mixture was incubated for 16 h at 4°C in the dark. Excess of SYBR gold was removed by using centrifugal filters Amicon – Centricon Plus 30kDa cut-off NMXL (MilliporeSigma, Burlington, MA, USA) and the fluorescence-labelled phages were concentrated by centrifugation at 2,000 x g during 1 h 30 at 4°C. Then, 300 µL of bacterial cultures at OD_600_ of 0.1 were infected with the labelled phages with a multiplicity of infection (MOI) equal to 400. After a 30 min-static incubation to allow phage adsorption, the cocultures were shaken at 180 rpm for one hour in the dark at 28°C. Then, they were centrifuged during 10 min at 4,300 x g and the pellets were resuspended in 20 µL of TSB. Imaging was performed with an Axioskop HBO 50W connected to a Axiocam 503 color camera (Zeiss, Oberkochen, Germany). Image acquisition was carried out with the manufacturer’s Zen software. Exposure time has been set at 0.03 s for natural light images and at 0.67s for fluorescent images (λEx = 495 nm, λEm = 537 nm). Phage adsorption was considered successful when a high proportion of cells were fluorescent. Three biological replicates were performed to confirm the observed phenotype.

**Pathogenicity assays of deletion and transposon knock-out mutants.** Six Φ*Xhv*-1-resistant mutants, including deletion and transposon insertion mutants in the *cps*, LPS1, LPS2, and LPS3 regions, were tested for virulence using both spray inoculation and infiltration assays on lettuce cv. Météore as described previously (22). Disease severity was measured every 2 days on each plant during three weeks using our scale disease index (22). In addition, infiltration assays were also conducted on lettuce leaves. Overnight bacterial cultures were spectrophotometrically adjusted to 0.2 OD_600_ (equivalent to 1.10^8^ CFU.mL^-1^) with sterile deionized water and subsequently serial-diluted to obtain a final suspension at 10^5^ CFU.mL^-1^. Leaves were perforated on each side of the midrib with sterile needles and infiltrated with 100 µL of the final bacterial suspension using a syringe. The bacterial population was enumerated at 0, 2, 7, and 10 days post inoculation (DPI) as described previously (23). At each time point, three infected leaves from three different plants were randomly collected and treated as independent technical replicates. Sampled leaves were surface sterilized in a 70% ethanol bath for 20 s and rinsed in sterile deionized H_2_O (dH_2_O) in order to count only the bacteria that had penetrated the leaf tissue. In all cases, 4 disks of 3.14 cm^2^ each were cut with a sterilized 1-cm-diameter punch on either side of the leaf midrib and pooled in 10 mL of sterile dH_2_O. Leaf disks were crushed using a T25 IKA Ultra-turrax disperser (IKA, Staufen im Bresigau, Germany) at full speed. The resulting leaf homogenates were serially diluted and plated onto 1/10^th^ TSA plates supplemented with cycloheximide at 50 µg.mL^-1^ with an easySpiral automated plater (Interscience, Saint-Nom-la-Bretèche, France). The plates were incubated at 28°C for 2 days and enumerated with a Scan 1200 automatic colony counter (Interscience). Bacterial populations were expressed in CFU.cm^-2^ of lettuce leaf.

**Motility assay.** The same set of six phage-resistant mutants tested in virulence assays was also assessed for twitching and swarming motility on TSB plates supplemented with 0.6 % or 1 % agar, respectively. Sucrose was added at a final concentration of 1 % to promote the expression of motility-associated genes (24). The OD_600_ of each bacterial culture was standardized to 0.8 and centrifuged at 5,432 x g for 10 min. Cell pellets were inoculated onto the middle of the plate with a sterile toothpick. Plates were incubated at 28°C during six days. A control with the wild type strain LM16734 was performed for each condition. The diameters of swarming and twitching zones were measured with ImageJ software (25). Experiments were performed three times and each treatment had three technical replicates.

**Data analysis and visualization.** All figures were generated using R (version 4.3.3; R Core Team) within RStudio (version 2023.12.1.402 “Ocean Storm” Release; RStudio Team), unless otherwise specified. Visualizations were primarily created using the ggplot2 package (version 3.5.1), with additional packages including readxl (version 1.4.3) for data import, dplyr (version 1.1.4) for data manipulation, ComplexHeatmap (version 2.18.0) and circlize (version 0.4.16) for specific heatmaps, and ggsignif (version 0.6.4) and gridExtra (version 2.3) for statistical annotations and figure arrangement, depending on the figure type. Adsorption kinetics, one-step growth curve, infiltration assay, disease progression in *planta* and motility assays were visualized using custom ggplot2 scripts, with summary statistics and transformations performed using dplyr. Statistical annotations (e.g., significance asterisks) were added with ggsignif. Heatmaps of bacterial defense systems and EOP values were generated with ComplexHeatmap using color gradients defined via the colorRamp2 () function from circlize. The phylogenetic tree generated using TYGS was aligned with the heatmap using Inkscape (version 1.4). The genomic map of phage Φ*Xhv*-1 was designed in SnapGene (version 7.2.1) and edited in Inkscape. TnSeq insertion profiles were produced with TRANSIT (version 3.2.1) and manually assembled into final layouts using Inkscape. All figures were exported in high-resolution JPEG and/or SVG formats (1200 dpi) using the ggsave () function.

All raw experimental data have been deposited on Zenodo (26) and are available at: <https://doi.org/10.5281/zenodo.15678039>

**REFERENCES**

1. Wommack KE, Williamson KE, Helton RR, Bench SR, Winget DM. Methods for the isolation of viruses from environmental samples. *In*: Clokie MRJ, Kropinski AM, éditeurs. Bacteriophages: Methods and Protocols, Volume 1: Isolation, Characterization, and Interactions. Totowa, NJ: Humana Press; 2009. p. 3‑14.

2. Meier-Kolthoff JP, Göker M. TYGS is an automated high-throughput platform for state-of-the-art genome-based taxonomy. Nat Commun. 16 mai 2019;10(1):2182.

3. Tesson F, Hervé A, Mordret E, Touchon M, d’Humières C, Cury J, et al. Systematic and quantitative view of the antiviral arsenal of prokaryotes. Nat Commun. 10 mai 2022;13(1):2561.

4. Gendre J, Ansaldi M, Olivenza DR, Denis Y, Casadesús J, Ginet N. Genetic mining of newly isolated Salmophages for phage therapy. Int J Mol Sci. 10 août 2022;23(16):8917.

5. Wingett SW, Andrews S. FastQ Screen: A tool for multi-genome mapping and quality control. F1000Res. 2018;7:1338.

6. Schmieder R, Edwards R. Quality control and preprocessing of metagenomic datasets. Bioinformatics. 15 mars 2011;27(6):863‑4.

7. Nurk S, Bankevich A, Antipov D, Gurevich AA, Korobeynikov A, Lapidus A, et al. Assembling single-cell genomes and mini-metagenomes from chimeric MDA products. J Comput Biol. oct 2013;20(10):714‑37.

8. Okonechnikov K, Golosova O, Fursov M, UGENE team. Unipro UGENE: a unified bioinformatics toolkit. Bioinformatics. 15 avr 2012;28(8):1166‑7.

9. Camacho C, Coulouris G, Avagyan V, Ma N, Papadopoulos J, Bealer K, et al. BLAST+: architecture and applications. BMC Bioinformatics. 15 déc 2009;10:421.

10. Langmead B, Salzberg SL. Fast gapped-read alignment with Bowtie 2. Nat Methods. 4 mars 2012;9(4):357‑9.

11. Li H, Handsaker B, Wysoker A, Fennell T, Ruan J, Homer N, et al. The Sequence Alignment/Map format and SAMtools. Bioinformatics. 15 août 2009;25(16):2078‑9.

12. Darling ACE, Mau B, Blattner FR, Perna NT. Mauve: multiple alignment of conserved genomic sequence with rearrangements. Genome Res. juill 2004;14(7):1394‑403.

13. Hockenberry AJ, Wilke CO. BACPHLIP: predicting bacteriophage lifestyle from conserved protein domains. PeerJ. 2021;9:e11396.

14. Borges R, Machado JP, Gomes C, Rocha AP, Antunes A. Measuring phylogenetic signal between categorical traits and phylogenies. Bioinformatics. 1 juin 2019;35(11):1862‑9.

15. Morinière L, Lecomte S, Gueguen E, Bertolla F. In vitro exploration of the *Xanthomonas hortorum* pv. *vitians* genome using transposon insertion sequencing and comparative genomics to discriminate between core and contextual essential genes. Microb Genom. sept 2019;7(6):000546.

16. Saavedra JT, Schwartzman JA, Gilmore MS. Mapping transposon insertions in bacterial genomes by arbitrarily primed PCR. Curr Protoc Mol Biol. 3 avr 2017;118:15.15.1-15.15.15.

17. DeJesus MA, Ambadipudi C, Baker R, Sassetti C, Ioerger TR. TRANSIT--A Software tool for Himar1 TnSeq analysis. PLoS Comput Biol. oct 2015;11(10):e1004401.

18. Boulanger A, Zischek C, Lautier M, Jamet S, Rival P, Carrère S, et al. The plant pathogen *Xanthomonas campestris* pv. *campestris* exploits N-acetylglucosamine during infection. mBio. 9 sept 2014;5(5):e01527-01514.

19. Xia Y, Li K, Li J, Wang T, Gu L, Xun L. T5 exonuclease-dependent assembly offers a low-cost method for efficient cloning and site-directed mutagenesis. Nucleic Acids Res. 20 févr 2019;47(3):e15.

20. Ditta G, Stanfield S, Corbin D, Helinski DR. Broad host range DNA cloning system for gram-negative bacteria: construction of a gene bank of *Rhizobium meliloti*. Proc Natl Acad Sci U S A. déc 1980;77(12):7347‑51.

21. Holtappels D, Fortuna KJ, Moons L, Broeckaert N, Bäcker LE, Venneman S, et al. The potential of bacteriophages to control *Xanthomonas campestris* pv. *campestris* at different stages of disease development. Microb Biotechnol. juin 2022;15(6):1762‑82.

22. Morinière L, Burlet A, Rosenthal ER, Nesme X, Portier P, Bull CT, et al. Clarifying the taxonomy of the causal agent of bacterial leaf spot of lettuce through a polyphasic approach reveals that *Xanthomonas cynarae* Trébaol *et al*. 2000 emend. Timilsina et al. 2019 is a later heterotypic synonym of *Xanthomonas hortorum* Vauterin *et al*. 1995. Syst Appl Microbiol. juill 2020;43(4):126087.

23. Morinière L, Mirabel L, Gueguen E, Bertolla F. A comprehensive overview of the genes and functions required for lettuce infection by the hemibiotrophic phytopathogen *Xanthomonas hortorum* pv. *vitians*. mSystems. 26 avr 2022;7(2):e0129021.

24. Tian T, Sun B, Shi H, Gao T, He Y, Li Y, et al. Sucrose triggers a novel signaling cascade promoting *Bacillus subtilis* rhizosphere colonization. The ISME Journal. 1 sept 2021;15(9):2723‑37.

25. Schneider CA, Rasband WS, Eliceiri KW. NIH Image to ImageJ: 25 years of image analysis. Nat Methods. juill 2012;9(7):671‑5.

26. Baud A. Dataset belonging to Unveiling host interactions and evolutionary constraints of a novel bacteriophage infecting *Xanthomonas hortorum* pv. *vitians*. *Zenodo*[10.5281/zenodo.15678038](https://doi.org/10.5281/zenodo.15678038) (2025).
