# Supplementary figures and images for: Unveiling Host Interactions and Evolutionary Constraints of a Novel Bacteriophage Infecting Xanthomonas hortorum pv. vitians
Source: Environ Microbiol Rep. 2025 Oct 30;17(6):e70171. doi: 10.1111/1758-2229.70171 (PMC12573098; doi:10.1111/1758-2229.70171)

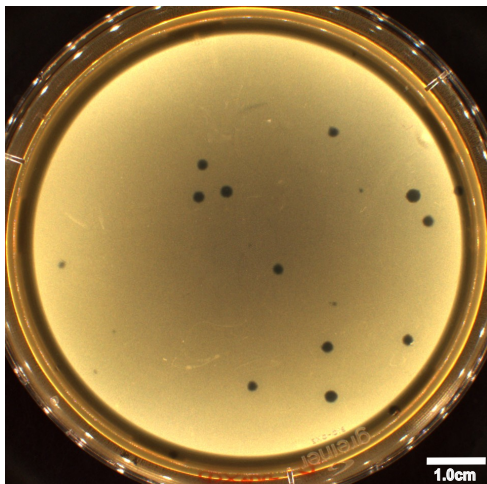

**Figure S1.**

Supplement: Supplementary file 2 — Figure S1: Plaque morphology of ΦXhv‐1 on a 0.6% soft agar TSB plate seeded with X. hortorum pv. vitians LM16734, forming circular and clear plaques. [file EMI4-17-e70171-s003.pdf]

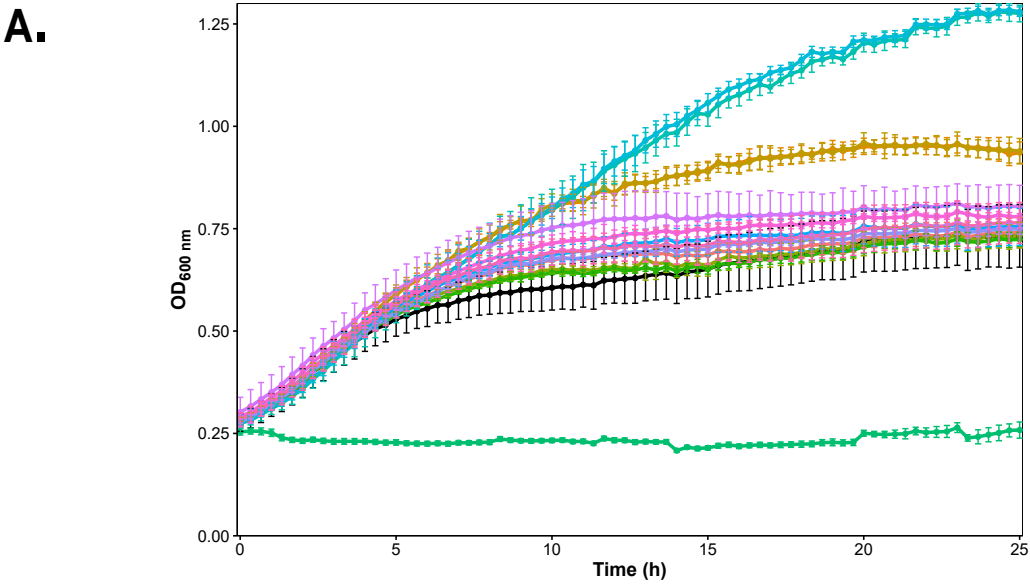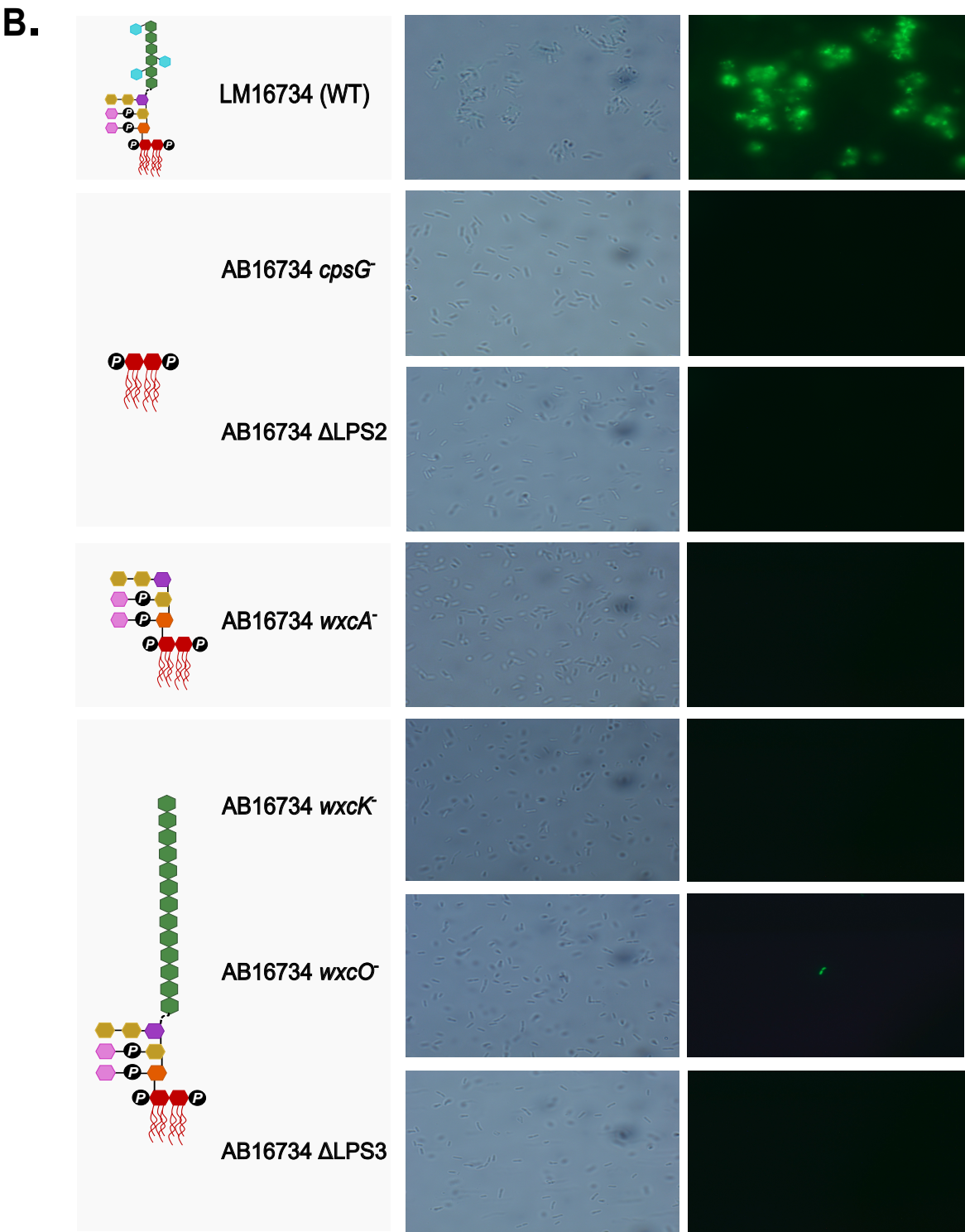

Figure S2.

Supplement: Supplementary file 3 — Figure S2: Phage ΦXhv‐1 resistance phenotype in mutants revealed by growth kinetics and fluorescence‐based adsorption dynamics. (A) Growth kinetics of wild‐type X. hortorum pv. vitians LM16734 and mutants in liquid culture, with and without ΦXhv‐1 infection. Optical density at 600 nm (OD600) was recorded every 20 min over 25 h. Each point represents the mean absorbance from five technical replicates, with error bars indicating the 95% confidence interval. While the wild‐type strain (black curve) showed significant growth inhibition, mutants exhibited sustained growth, confirming their resistance to ΦXhv‐1. (B) Fluorescence microscopy analysis of phage adsorption to bacterial cells. Wild‐type and transposon mutants were infected with ΦXhv‐1 at a multiplicity of infection (MOI) of 400 using SYBR Gold‐stained ΦXhv‐1 particles. After incubation and washing, images were acquired under natural light (left) and fluorescence (right) conditions (λEx = 495 nm, λEm = 537 nm). Strong fluorescence in the wild‐type strain indicates efficient phage adsorption, while mutants exhibited significantly reduced fluorescence, suggesting impaired phage binding or entry. [file EMI4-17-e70171-s004.pdf]
