# Supplementary material for: Unveiling Host Interactions and Evolutionary Constraints of a Novel Bacteriophage Infecting Xanthomonas hortorum pv. vitians
Source: Environ Microbiol Rep. 2025 Oct 30;17(6):e70171. doi: 10.1111/1758-2229.70171 (PMC12573098; doi:10.1111/1758-2229.70171)
Supplement: Supplementary file 4 — Table S1: List of bacterial strains and plasmids used or generated in this study. Table S2: List of primers used in this study. Sequences corresponding to restriction sites are underlined and sequences non‐homologous to Xhv are represented in lowercase and italics. Table S3: Transposon‐insertion sequencing (Tn‐seq) statistics before Totreads normalisation. Table S4: Predicted coding sequences and functional annotations of the ΦXhv‐1 genome. [file EMI4-17-e70171-s001.docx]

**Table S1. List of bacterial strains and plasmids used or generated in this study.**

| **Bacterial strain** | **Host of isolation** | **Geographic origin** | **Year of isolation** | **MLSA** | **Genotype and relevant characteristics^a^** | **Accesion number on GenBank** | **References** | **Used for** |
| --- | --- | --- | --- | --- | --- | --- | --- | --- |
| ***Xanthomonas hortorum* pv. *vitians*** |  |  |  |  |  |  |  |  |
| CFBP8638 (= LM16734) | *Lactuca sativa* cv. Parinice | Savoie, France | 2016 | A | wild type, isolation and amplification strain of ф*Xhv*-1 / Amp^R^ ; Kan^S^ | GCA_014338485.1 | Morinière *et al*., 2020 | All experiments |
| CFP498 | *Lactuca sp.* | United States | 1949 | A | Amp^R^ | GCA_012922255.1 | Vauterin *et al*., 1995 | Host range |
| CFBP499 | *Lactuca scariola* | United States | 1961 | B | Amp^R^ | GCA_012922335.1 | Vauterin *et al*., 1995 | Host range |
| CFBP3978 | *Lactuca sativa* | France | 1994 | C | Amp^R^ | GCA_012922195.1 | Vauterin *et al*., 1995 | Host range |
| CFBP7999 | *Solanum lycopersicum* | Gisborne, New Zealand | 1980 | C | Amp^R^ | GCA_001908775.1 | Jones *et* *al*., 2004 | Host range |
| CFBP8639 | *Lactuca sativa* cv. Almagro | Savoie, France | 2016 | C | Amp^R^ | GCA_012922125.1 | Morinière *et al*., 2020 | Host range |
| CFBP8640 | *Lactuca sativa* cv. Minestrone | Isère, France | 2016 | B | Amp^R^ | GCA_012922175.1 | Morinière *et al*., 2020 | Host range |
| CFBP8641 | *Lactuca sativa* | Ain, France | 2016 | C | Amp^R^ | GCA_027887235.1 | Morinière *et al*., 2020 | Host range |
| CFBP8642 | *Lactuca sativa* cv. Funride | Rhône, France | 2016 | A | Amp^R^ |  | Morinière *et al*., 2020 | Host range |
| CFBP8643 | *Lactuca sativa* cv. Corbana | Savoie, France | 2016 | B | Amp^R^ |  | Morinière *et al*., 2020 | Host range |
| CFBP8686 | *Lactuca sativa* | Zimbabwe | 1966 | C | Amp^R^ | GCA_021352975.1 | Vauterin *et al*., 1995 | Host range |
| LM16736 | *Lactuca sativa* cv. Almagro | Savoie, France | 2016 | A | Amp^R^ |  | Morinière *et al*., 2020 | Host range |
| LM17697 | *Lactuca sativa* cv*.* Julena | Rhône, France | 2017 | A | Amp^R^ |  | Morinière *et al*., 2020 | Host range |
| AB16734 *cpsG*^-^ |  |  |  | A | CFBP8638 with transposon insertion in *cpsG. cpsG*::TnHimar1 / Kan^R^ |  | This study | Virulence, motility |
| AB16734 *wxcA*^-^ |  |  |  | A | CFBP8638 with transposon insertion in *wxcA. wxcA*::TnHimar1 / Kan^R^ |  | This study | Virulence, motility |
| AB16734 *wxcK*^-^ |  |  |  | A | CFBP8638 with transposon insertion in *cpsG. wxcK*::TnHimar1 / Kan^R^ |  | This study | Virulence, motility |
| AB16734 *wxcO*^-^ |  |  |  | A | CFBP8638 with transposon insertion in *cpsG. wxcO*::TnHimar1 / Kan^R^ |  | This study | Virulence, motility |
| AB16734 ∆LPS3 |  |  |  | A | CFBP8638 with a deletion by homologous recombination ∆(*wxcK*-*wxc*O) / Amp^R^ ; Kan^S^ |  | This study | Virulence, motility |
| AB16734 ∆LPS2 |  |  |  | A | CFBP8638 with a deletion by homologous recombination ∆(*rmd*-*gmd*) / Amp^R^ ; Kan^S^ |  | This study | Virulence, motility |
| ***Escherichia coli*** |  |  |  |  |  |  |  |  |
| DH5α |  |  |  |  | F^-^ ф80 ∆*lacZ*∆M15 ∆(*IacZYA*-*argF*)*U169* *recA1 endA1 hsdRJ7* (r_K_^-^ m_K_^+^ ) *supE44 thi-1* lambda *gyrA96 relA1* |  | Hanahan *et al*., 1983 |  |
| HB101 |  |  |  |  | *recA hsdR hsdM strA pro leu thi* |  | Boyer and Roulland-Dussoix, 1969 |  |
| **Plasmids** | **Genotype and relevant characteristics** | | | | | | **References** |  |
| RK600 | *ori-ColE1oriV* RP4*tra^+^* RP4*oriT* helper plasmid in triparental matings / Cm^R^ | | | | | | Kessler *et al*., 1992 |  |
| pK18*mobsacB* | pMB1 *ori-ColE1* *mob*+ *sacB*+ / Kan^R^ | | | | | | Schäfer *et al*., 1994 |  |
| pK18*mobsacB* ∆LPS3 | pK18*mobsacB* derivative with two fragment cloned into the EcoRI and HindIII site. A 538 bp fragment corresponding to the region upstream of the start codon of XHV734_4236 and a 809 bp fragment corresponding to the region downstream of the stop codon XHV734_4240 / Kan^R^ | | | | | | This study |  |
| pK18*mobsacB* ∆LPS2 | pK18*mobsacB* derivative with two fragment cloned into the EcoRI and HindIII site. A 772 bp fragment corresponding to the region upstream of the start codon of XHV734_4241 and a 763 bp fragment corresponding to the region downstream of the stop codon XHV734_4242 / Kan^R^ | | | | | | This study |  |
| **^a^** Amp^R^, Kan^R^, Cm^R^ indicate resistance to ampicillin, kanamycin and chloramphenicol, respectively. | | | | |  |  |  |  |

**Table S2. List of primers used in this study.** Sequences corresponding to restriction sites are underlined and sequences non homologous to *Xhv* are represented in lowercase and italics.

| **Molecular objectives** | **Number / name of primer** | **Targeted locus** | **Sequence (from 5' to 3')** | **Tm (°C)** | **Ta (°C)** | **Amplification size (bp)** |
| --- | --- | --- | --- | --- | --- | --- |
| **Determination of the transposon insertion site  (AP-PCR)** | 2012 / Himar1_F1 | *nptII* of transposon Himar1 | CCTGCCGAGAAAGTATCCATCA | 59 | 30 | Variable |
|  | 2013 / AP_R1 | Random | ATGCCACGAGTCGACTAGTACNNNNNNNNNNACGCC | - |  |  |
|  | 2016 / Himar1_F2 | Transposon Himar1 downstream of 2012 | TCCTGACGGATGGCCTTTTTGC | 61 | 55 | Variable |
|  | 2014 / AP_R2 | Random | ATGCCACGAGTCGACTAGTAC | 60.7 |  |  |
|  |  |  |  |  |  |  |
| **Construction of deletion mutants by homologous recombination** | 2025 / ΔLPS3_upstreamF_overlap | XHV734_4235 *etfA* | *aggaaacagctatgacatgattacgaattc*GATTTGTTCTCGCTGCTTCCG | 59 | 54 | 538 |
|  | 2022 / ΔLPS3_upstreamR | upstream of the ATG of XHV734_4236 *wxcK* | *aatgcctggtatttttgttcggcg*TGGGATCCGCAGAGTTTGAG | 60 |  |  |
|  | 2023 / ΔLPS3_downstreamF | 73 bp upstream of XHV734_4241 *rmd* | *catctactcaaactctgcggatccc*ACGCCGAACAAAAATACCAG | 55 | 50 | 809 |
|  | 2024 / ΔLPS3_downstreamR_overlap | XHV734_4241 *rmd* | *cgttgtaaaacgacggccagtgccaagctt*TTTTTACCGGGTCAATCTGCT | 56 |  |  |
|  | 2042 / ∆LPS2_upstream F | XHV734_4240 *wxcO* | *aacagctatgacatgattacgaattc*AGTGATTGCCGAATGGACA | 55 | 51 | 772 |
|  | 2043 / ∆LPS2_upstream R | 39 bp upstream of XHV734_4241 *rmd* | *atcggctcaaagccggaaccaata*CGGAAAATCCAGCCGAAGTCT | 57 |  |  |
|  | 2044 / ∆LPS2_downstream F | 19 bp downstream of XHV734_4242 *gmd* | *aggcgagacttcggctggattttc*CGTATTGGTTCGGCTTTGAG | 58 | 53 | 763 |
|  | 2041 / ∆LPS2_downstreamR | XHV734_4244 *wxcD* | *taaaacgacggccagtgccaagctt*AGTAGAAGCCGCCCAAGATT | 54 |  |  |
|  |  |  |  |  |  |  |
| **Verification of the TEDA  in pK18*mobsacB*** | 2021 / pK18_TEDA_F | 168 bp upstream of lacZα | AGCTGGCACGACAGGTTTC | 60 | 52 | pK18*mobsacB* empty = 406 pK18*mobsacB*∆LPS3 = 1598 pK18*mobsacB*∆LPS2 = 1795 |
|  | 2026 / pK18_TEDA_R | *lacZ*α | AGCTAGCTTATCGCCATTCG | 57 |  |  |
|  |  |  |  |  |  |  |
| **Verification of crossing-over (CO) in *Xhv* CFBP8638** | G1680 / SpéXhv_F | XHV734_5023 | GCCAGAAAAGCAGGTTTGGA | 58.4 | 55 | 458 |
|  | G1681 / SpéXhv_R | XHV734_5023 | TATCAATGGTCTGGCTCCCC | 60.5 |  |  |
|  | 2035 / nptII_F | *nptII* of pK18*mobsacB* | ACTGAAGCGGGAAGGGACTG | 61 | 57 | 154 |
|  | 2036 / nptII_R | *nptII* of *pK18mobsacB* | GGTGGTCGAATGGGCAGGTA | 60 |  |  |
|  | 2040 / AB16734ΔLPS3_2CO_F | XHV734_4235 *etfA* | CCGAGAATTTCCAGCACATC | 56 | 51 | Mutant = 1805 Wild type = 7646 |
|  | 2039 / AB16734ΔLPS3_2CO_R | XHV734_4242 *gmd* | CCGATCTGAAGAGGAACGAG | 57 |  |  |
|  | 2047 / AB16734ΔLPS2_2CO_F | XHV734_4240 *wxcO* | CTTGCACGCATATGACCTTG | 56 | 51 | Mutant = 1779 Wild type = 3794 |
|  | 2048 / AB16734ΔLPS2_2CO_R | XHV734_4244 *wxcD* | CCGATGTCTTCTGCAGTTCA | 54 |  |  |

**Table S3. Transposon-insertion sequencing (Tn-seq) statistics before TTR normalization.**

| Replicate | Sequencing yield | No. of Tn-end containing reads | Read count (unique TA sites) | TA hits^a^ | Insertion density^a^ | Mean read count over non-zero TA^b^ |
| --- | --- | --- | --- | --- | --- | --- |
| **TnA** | 20 326 655 | 17 979 869 | 17 848 943 | 58 171 | 0.682 | 306.8 |
| **TnB** | 29 435 946 | 26 310 315 | 26 154 950 | 60 146 | 0.705 | 434.9 |
| **P1A** | 27 576 142 | 25 942 932 | 25 842 896 | 54 618 | 0.640 | 473.2 |
| **P1B** | 26 593 521 | 25 389 175 | 25 389 175 | 53 570 | 0.628 | 472.3 |
| ^a^ *X. hortorum* pv. *vitians* LM16734 chromosome contains 85,314 TA sites. Insertion density is calculated as the fraction of TA sites with at least one read mapped over total number of TA sites. ^b^ Mean read count per TA site containing at least one read. | | | | | | |

**Table S4. Predicted coding sequences and functional annotations of the ΦXhv-1 genome.**

| **Locus_tag** | **Start** | **End** | **Size (bp)** | **Strand** | **Predicted function** |
| --- | --- | --- | --- | --- | --- |
| Xhv1_0001 | 27 | 497 | 471 | + | Terminase, Small subunit |
| Xhv1_0002 | 497 | 1,957 | 1,461 | + | Phage terminase, large subunit |
| Xhv1_0003 | 2,007 | 3,617 | 1,611 | + | Portal protein |
| Xhv1_0004 | 3,592 | 4,488 | 897 | + | Phage minor capsid protein |
| Xhv1_0005 | 4,451 | 5,215 | 765 | - | DNA modification methyltransferase |
| Xhv1_0006 | 5,319 | 6,446 | 1,128 | + | Prohead core protein protease |
| Xhv1_0007 | 6,471 | 6,995 | 525 | + | Capsid fiber protein |
| Xhv1_0008 | 7,023 | 8,03 | 1,008 | + | Major Capsid Protein |
| Xhv1_0009 | 8,081 | 9,112 | 1,032 | - | DNA-cytosine methyltransferase |
| Xhv1_0010 | 9,073 | 9,27 | 198 | - | DnaK suppressor protein |
| Xhv1_0011 | 9,647 | 10,057 | 411 | + | Phage protein |
| Xhv1_0012 | 10,07 | 10,576 | 507 | + | Phage protein |
| Xhv1_0013 | 10,573 | 11,085 | 513 | + | Phage protein |
| Xhv1_0014 | 11,202 | 11,651 | 450 | + | Phage protein |
| Xhv1_0015 | 11,651 | 12,181 | 531 | + | Tail terminator protein |
| Xhv1_0016 | 12,208 | 13,695 | 1,488 | + | Sheath protein |
| Xhv1_0017 | 13,708 | 14,139 | 432 | + | Putative phage XkdM-like protein |
| Xhv1_0018 | 14,609 | 15,439 | 831 | - | Phage protein |
| Xhv1_0019 | 15,436 | 15,741 | 306 | - | Repressor protein |
| Xhv1_0020 | 15,771 | 16,205 | 435 | - | Phage protein |
| Xhv1_0021 | 16,202 | 16,348 | 147 | - | Replicative DNA helicase |
| Xhv1_0022 | 16,345 | 16,725 | 381 | - | Hypothetical protein |
| Xhv1_0023 | 16,736 | 16,999 | 264 | - | Hypothetical protein |
| Xhv1_0024 | 17,002 | 17,169 | 168 | - | Hypothetical protein |
| Xhv1_0025 | 17,232 | 19,205 | 1,974 | - | DNA polymerase I, phage-associated |
| Xhv1_0026 | 19,277 | 19,801 | 525 | - | Hypothetical protein |
| Xhv1_0027 | 19,906 | 21,726 | 1,821 | - | DNA helicase, phage-associated |
| Xhv1_0028 | 21,811 | 22,029 | 219 | - | Hypothetical protein |
| Xhv1_0029 | 22,05 | 22,184 | 135 | - | Hypothetical protein |
| Xhv1_0030 | 22,493 | 22,903 | 411 | - | Phage protein |
| Xhv1_0031 | 22,9 | 23,088 | 189 | - | Hypothetical protein |
| Xhv1_0032 | 23,085 | 23,237 | 153 | - | Hypothetical protein |
| Xhv1_0033 | 23,234 | 23,386 | 153 | - | Hypothetical protein |
| Xhv1_0034 | 23,379 | 23,519 | 141 | - | Hypothetical protein |
| Xhv1_0035 | 23,581 | 23,799 | 219 | - | Putative Double-stranded RNA-specific adenosine deaminase |
| Xhv1_0036 | 23,796 | 23,975 | 180 | - | Transcription initiation factor |
| Xhv1_0037 | 23,972 | 24,412 | 441 | - | Hypothetical protein |
| Xhv1_0038 | 24,402 | 24,587 | 186 | - | Hypothetical protein |
| Xhv1_0039 | 24,584 | 25,072 | 489 | - | Hypothetical protein |
| Xhv1_0040 | 25,069 | 25,338 | 270 | - | Phage protein |
| Xhv1_0041 | 25,335 | 25,622 | 288 | - | Phage protein |
| Xhv1_0042 | 25,619 | 25,783 | 165 | - | Hypothetical protein |
| Xhv1_0043 | 25,87 | 26,118 | 249 | - | Hypothetical protein |
| Xhv1_0044 | 26,115 | 26,342 | 228 | - | Hypothetical protein |
| Xhv1_0045 | 26,418 | 28,907 | 2,49 | - | DNA primase, phage associated # P4-type |
| Xhv1_0046 | 28,937 | 29,257 | 321 | - | Hypothetical protein |
| Xhv1_0047 | 29,59 | 29,973 | 384 | + | Baseplate wedge protein |
| Xhv1_0048 | 29,97 | 30,383 | 414 | + | Phage protein |
| Xhv1_0049 | 30,453 | 31,172 | 720 | + | Phage tail fiber protein |
| Xhv1_0050 | 31,289 | 31,828 | 540 | + | Phage protein |
| Xhv1_0051 | 31,825 | 32,235 | 411 | + | Phage protein |
| Xhv1_0052 | 32,24 | 32,926 | 687 | + | Putative Ripcord PA0626 protein |
| Xhv1_0053 | 32,928 | 34,799 | 1,872 | + | Tail length tape measure protein |
| Xhv1_0054 | 34,796 | 35,695 | 900 | + | Contractile injection system protein |
| Xhv1_0055 | 35,679 | 35,93 | 252 | + | Hypothetical protein |
| Xhv1_0056 | 35,914 | 36,633 | 720 | + | Phage baseplate protein; Cell Puncturing Device |
| Xhv1_0057 | 36,651 | 37,919 | 1,269 | + | Baseplate protein |
| Xhv1_0058 | 37,922 | 38,605 | 684 | + | Baseplate wedge protein |
| Xhv1_0059 | 38,616 | 40,013 | 1,398 | + | Putative tail fiber protein |
| Xhv1_0060 | 40,08 | 40,253 | 174 | - | Hypothetical protein |
| Xhv1_0061 | 40,257 | 40,541 | 285 | - | Hypothetical protein |
| Xhv1_0062 | 40,534 | 41,172 | 639 | - | Methyl-accepting chemotaxis protein I (serine chemoreceptor protein) |
| Xhv1_0063 | 41,162 | 41,557 | 396 | - | Phage holin |
| Xhv1_0064 | 41,647 | 42,204 | 558 | - | Membrane-bound lytic murein transglycosylase D precursor |
| Xhv1_0065 | 42,201 | 42,566 | 366 | - | Phage protein |
| Xhv1_0066 | 43,673 | 44,317 | 645 | - | Phage protein |
| Xhv1_0067 | 44,374 | 44,574 | 201 | - | Hypothetical protein |
| Xhv1_0068 | 44,574 | 45,86 | 1,287 | - | ATP-dependent DNA helicase |
